# Research on sugarcane stem counting method based on improved RT-DETR-seg

**DOI:** 10.3389/fpls.2026.1893319

**Published:** 2026-07-29

**Authors:** Xiangwu Deng, Yuanjia Ma, Hanhong Zheng

**Affiliations:** College of Electronic Information Engineering, Guangdong University of Petrochemical Technology, Maoming, China

**Keywords:** dense occlusion, instance segmentation, RT-DETR-seg, stem counting, sugarcane

## Abstract

**Introduction:**

Sugarcane is an important sugar crop. In the life cycle management of sugarcane, counting serves as the core foundational data for yield estimation, precise water and fertilizer regulation, and the optimization of intelligent harvesting equipment. Therefore, accurate counting of sugarcane in the field facilitates yield prediction and fine-grained management of water and fertilizer resources.

**Methods:**

To address the issue of instance feature fragmentation caused by the elongated shape of sugarcane and complex occlusions, this paper presents an improved RT-DETR-seg (Real-Time Detection Transformer instance segmentation) model. The specific improvements include: adding asymmetric strip convolution (ASCs) to the backbone network to enhance the perception of elongated sugarcane targets through vertical feature aggregation, while also suppressing background noise and embedding a coordinate attention (CA) mechanism into the hybrid encoder to leverage long-range spatial dependencies, thereby bridging occluded regions and establishing feature associations to effectively mitigate repeated counts caused by steam leaf truncation. Furthermore, a joint loss function named the moment-gradient regularized (MGR) loss is designed, which integrates spatial moments and gradient field constraints and incorporates an aspect ratio penalty and edge smoothness constraints to significantly increase the segmentation accuracy under strong lighting and mottled shadow conditions.

**Results:**

Ablation experiments were conducted to individually verify the performance improvements associated with the three major enhancement modules: the ASC, CA mechanism, and MGR loss function. Compared with mainstream single-stage instance segmentation algorithms, the detection performance and counting advantages of the improved model in sugarcane scenes with dense occlusions are verified. The experimental results revealed that the improved model achieved a bounding box detection accuracy of 91.03% and a mask segmentation accuracy of 81.04% on the self-constructed multi-scenario sugarcane dataset, whereas the mean absolute error (MAE) of the counting results decreased significantly from 1.09 with the baseline model to 0.28 with the improved model.

**Discussion:**

This study presents a sugarcane counting method based on an improved RT-DETR-seg model to address the challenge of occlusion in complex sugarcane field environments, ultimately enabling high-precision, high-throughput automated monitoring of slender economic crops such as sugarcane. The enhanced algorithm balances robust perception and real-time inference under complicated field conditions, providing a solid algorithmic foundation for the development and deployment of edge-side counting systems.

## 1 Introduction

Sugarcane, as a key global sugar crop and raw material for renewable energy systems, plays a strategic role in ensuring sugar security, optimizing energy structures, and promoting agricultural economic growth (Deng et al., 2024). In China, sugarcane cultivation is concentrated mainly in southern regions (Guangdong Province, Guangxi Province, and Hainan Province), which are the core areas for sugar production (Luo et al., 2026; Hu et al., 2025). As this crop is closely related to the national economy and people’s livelihoods, the efficient development of the sugarcane industry directly affects the stability of the food industry and increase in farmer incomes. Moreover, sugarcane resource utilization is crucial for promoting the development of renewable energy systems and achieving “carbon neutrality” goals (Pan et al., 2024). In the management of the lifecycle of sugarcane, sugarcane counting (SC) serves as the core data for estimating sugarcane production, precisely regulating water and fertilizer (Zhao et al., 2026), and optimizing intelligent harvesting equipment; thus, SC directly determines the scientific validity of field management and the economic efficiency of harvesting operations (Zhu et al., 2026; Yang et al., 2024).

Traditional SC methods rely heavily on manual operation, which has disadvantages such as high labor intensity and low efficiency. A single person can complete only approximately 0.3 hectares of counting tasks per day (Lu et al., 2010). Especially in complex field scenarios during the tillering stage where plants are densely packed (with a plant spacing of 5–10 cm) and stem nodes are severely obstructed in the middle and later stages, the error rate of manual statistics can reach as high as 20%–30% (Chen et al., 2023), and is the error rate also affected by the subjective experience of the counting staff, making it impossible to provide reliable support for precision agriculture (Meng et al., 2019). With the intensification of agricultural labor shortages and rising costs (He et al., 2016), integrating robot vision and deep learning technologies to extract high-resolution image features for efficient, noncontact automatic detection and counting has become the mainstream approach to overcome this bottleneck (Li et al., 2025b; Wang C.Y. et al., 2020; Yu et al., 2026).

The efficient identification and counting of crop targets are prerequisites for implementing field management strategies in precision agriculture. Early visual algorithms relied mainly on manually designed features, which were susceptible to factors such as lighting and dense occlusions (DOs) between targets, leading to insufficient robustness. In terms of traditional image processing methods, researchers have attempted to extract the contours of sugarcane stem (SS) nodes by using methods such as grey filtering, spatial projection, and mathematical morphology. They achieved certain results in controlled environments (Chen et al., 2021; Lowe, 2004). However, in the case of unstructured real farmland scenes, these methods have weak adaptability. Owing to the limitations of fixed thresholds and single visual features, these methods are easily disturbed by complex backgrounds and lighting changes; especially in the middle and late stages of sugarcane growth, the canopy severely overlaps, and traditional operators often produce broken or false edges, resulting in unstable detection results and making it difficult to support large-scale and high-throughput field operations (Jiang et al., 2021).

Traditional manually designed features are commonly affected by the field environment and have a relatively weak ability to adapt to the environment (Deng et al., 2018). To address the limitations of handcrafted features, current research has shifted towards the use of end-to-end automatic feature extraction methods, particularly when deep learning techniques are applied. In vision-related research in agriculture, deep learning methods such as convolutional neural networks (CNNs) have gradually been introduced. CNNs can automatically learn image features through end-to-end training (Kamilaris and Prenafeta-Boldú, 2018) and generally achieve robust recognition performance under varying field illumination conditions, complex backgrounds, and significant differences in crop morphology (Li H. et al., 2025). For deep learning-based target detection in field crop scenarios, YOLO series object detection models have been applied to agricultural tasks such as apple recognition (Tian et al., 2019); and maize tassel detection (Lu et al., 2015). In terms of sugarcane target detection, Li et al. (2025a) used an improved YOLOv8 model to detect local sugarcane stem nodes in the middle and lower parts of the sugarcane in sugarcane harvester scenes. The experimental results revealed that the improved YOLOv8 model had a P value of 96.8%. Studies have also shown that YOLO series models can mitigate the effects of weed occlusion and illumination variation to some extent (Bochkovskiy et al., 2020). In particular these, YOLOv4 achieves a favorable balance between detection accuracy and inference speed, making it suitable for use with agricultural robots in real-time field detection scenarios (Zhang L. et al., 2018). Moreover, the clustered nature of sugarcane growth can lead to severe occlusion between sugarcane targets and between sugarcane and sugarcane leaves in the same area. Compared with the other fruits and crops mentioned above, it is more difficult to create independent and continuous rectangular box boundaries for sugarcane targets. If a target box-based detection algorithm is used to label sugarcane, substantial interference information is present in the marked box when the area is manually identified. These factors are unfavorable for the model to learn the overall target features of sugarcane. Therefore, detection algorithms based on target boxes are not suitable for sugarcane detection and counting. Furthermore, for high-density, thin-stem crops such as sugarcane, issues such as large aspect-ratio variations among stems and mutual occlusions remain difficult to resolve effectively using YOLO series object detection algorithms (Sapkota et al., 2026). Since the counting problem for high-density thin-stem crops such as sugarcane is similar to that of dense crowd counting, the scale-adaptive network structures developed in the crowd counting domain can provide reference information for improving dense SS counting methods (Machefer et al., 2020).

Although object detection algorithms have made progress in crop recognition tasks, their primary output consists of rectangular bounding boxes. For high-density, highly occluded, thin SSs, the detected bounding boxes inevitably include background pixels or local features of adjacent stems within the box. Owing to the relatively coarse spatial localization of bounding box-based object detection algorithms, during the nonmaximum suppression (NMS) postprocessing stage, the algorithm may mistakenly classify truly overlapping targets as redundant boxes and filter them out, leading to omissions in the counting results. Consequently, continuing to rely on rectangular bounding boxes for SC makes it difficult to thoroughly address the issues of deduplication and missed detection in high-density SSs scenes. To meet the demand for accurate identification and counting of SSs in the field, current research must move beyond coarse-grained detection frameworks and introduce instance segmentation techniques capable of pixel-level discrimination to restructure the target counting logic.

Instance segmentation, as a semantic perception task in computer vision, fundamentally involves the pixel-level semantic discrimination of targets and the precise separation of individual spatial locations. To overcome the problem of counting dense SSs in the field, the visual task needs to be advanced from target detection to Instance segmentation. The Mask R-CNN architecture (Machefer et al., 2020) has been utilized to achieve pixel-level mask segmentation of individual plants in scenes with complex backgrounds. In environments with high crop density and severe overlap, mask segmentation methods can effectively isolate adjacent stems and reduce the problems of missed detection and incorrect detection caused by overlapping detection boxes. Therefore, the use of instance segmentation methods for the identification and counting of dense crops has good practical application value.

Early Instance segmentation research mostly relied on CNNs as the feature extraction backbone. For instance, Mask R-CNN was proposed, which involved “segmentation guided by detection” two-stage standard process, thereby providing the technical foundation for this field (He et al., 2017). Subsequently, to meet the real-time engineering requirements of agricultural equipment, YOLACT (Bolya et al., 2019) and YOLO series (Liu et al., 2026) single-stage network models were developed, significantly increasing the mask inference efficiency. Owing to the limitations of the convolution operators in CNNs with respect to the local receptive field, under complex working conditions, such as the intersection of slender crops or the lateral obstruction of targets by broad and withered leaves, the network often fails to establish context associations across the blocked areas, ultimately resulting in feature breaks and fragmentation in the output predicted masks. When such CNN architectures are directly applied to unstructured large-field environments, the limitations of their feature extraction mechanisms gradually become apparent.

To overcome the perceptual defects caused by the local field of view of CNNs, a visual architecture based on transformers represents a new approach for target segmentation in complex scenes. Represented by the Swin Transformer (Liu et al., 2021) and SegFormer (Xie et al., 2021), this type of architecture relies on the self-attention mechanism, which can effectively capture the global spatial dependencies across regions while maintaining reasonable computational costs. The robustness of the transformer architecture was verified t in the task of weed and crop classification using high-resolution images collected by unmanned aerial vehicles (Reedha et al., 2022), further expanding the application range of this technology in precision agriculture. Thus, even if local parts of the crop are severely obstructed, the model can still maintain the semantic coherence of the target individual by using global context information. Moreover, the end-to-end architecture has evolved. A detection transformer (DETR) series (Carion et al., 2020) was proposed, which introduced set prediction and bipartite graph matching mechanisms, enabling the network to directly output nonredundant prediction results, thereby completely removing the NMS step, which is prone to causing false deletions in dense scenes.

By integrating the advantages of the two aforementioned technical approaches, real-time global perception architecture such as RT-DETR were proposed (Lv et al., 2024). This model retains the global modelling characteristics of the transformer while outperforming convolutional networks of the same level in terms of the inference speed. In recent years, cutting-edge segmentation algorithms such as SOLO (Wang X. et al., 2020), Mask2Former (Cheng et al., 2022), and large SAM-based models (Kirillov et al., 2023) have been proposed in the visual field. Considering the frequent physical occlusion issues in real farmland scenes and practical engineering constraints, such as limited hardware computing power at the edge, the real-time instance segmentation architecture based on RT-DETR-seg (Real-Time Detection Transformer instance segmentation) achieves a good balance between accuracy and speed.

Although deep learning techniques have shown considerable application potential, SSs in unstructured farmland environments are characterized by a high-density distribution and severe physical occlusions. Previous farmland vision detection methods have mostly employed two-stage networks that rely on region proposals such as Mask R-CNN or single-stage segmentation models based on the YOLO series. However, such CNNs are heavily dependent on NMS to filter overlapping prediction boxes at the inference end. In highly overlapping sugarcane environments, the intersection over union (IoU) threshold of the NMS strategy can easily result in erroneous deletions, with real stems identified as redundant boxes and removed. Thus, SS counting under complex field conditions still faces significant challenges.

To fundamentally address the above limitations at the algorithmic level, in this paper, the transformer-based end-to-end RT-DETR-seg model is selected as the baseline algorithm. This study focuses on field SS counting and instance segmentation methods, with the goal of improving the end-to-end deep learning architecture to address the issues of large aspect-ratio variations and mutual occlusion among SSs, ultimately achieving high-precision, high-throughput automated monitoring of elongated economic crops such as SS. The specific research content is as follows:

1) Targeted improvements are applied to the RT-DETR seg model. To fit the slender longitudinal characteristics of sugarcane, asymmetric strip convolutions (ASCs) were introduced into the backbone network. 2) By embedding a coordinate attention (CA) mechanism into the model, the model’s anti-occlusion ability is enhanced, and the spatial correlation relationships among the targets are established. 3) We designed the MGR joint loss function to address the problem of uneven sugarcane edge segmentation under complex lighting conditions, effectively increasing the accuracy and robustness of the model in field scenarios.

## 2 Materials and methods

### 2.1 Sugarcane image acquisition and processing

Compared with those in common scenarios such as urban or indoor environments, the visual characteristics of unstructured agricultural environments in sugarcane fields significantly differ. Taking the sugarcane field inspection in this study as an example, the sugarcane plants are slender and densely distributed, and severe obstruction by dry leaves and overlapping stems is common during the later stages of growth. In addition, the real sugarcane farming environment is accompanied by variable lighting conditions (such as strong direct sunlight, backlighting, and tree crown shadows) and background interference from soil and weeds. General dataset lack such specific environmental samples, making it difficult to directly support the high-precision recognition and counting of sugarcane.

**Figure 1:**
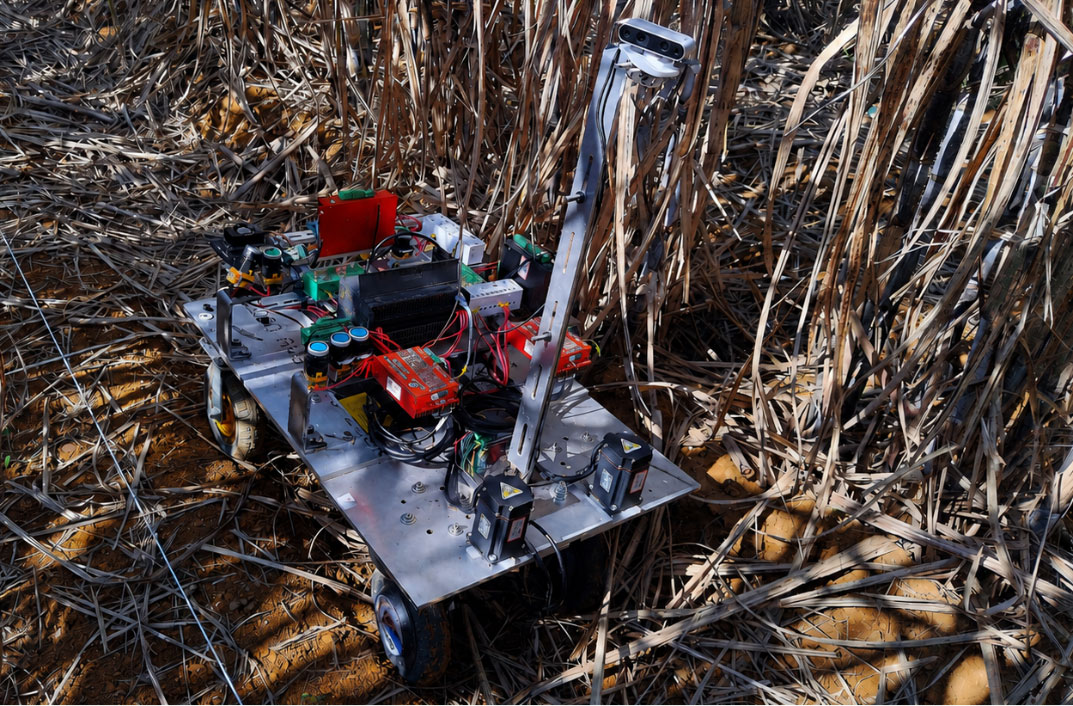
Physical diagram of the inspection robot experimental platform.

This study is grounded in the complex field environment of sugarcane. Sugarcane images were collected at a sugarcane field (Tan Village, Jintang Town, Maonan District, Maoming City, Guangdong Province), and an inspection robot platform was used to acquire a field dataset during the mid-to-late growth stages of the sugarcane. A visual acquisition device, namely, D435I stereo camera was rigidly mounted on top of the robot at approximately 75 cm above the ground (**Figure 1**), maintaining a slightly upwards shooting angle. The resolution of the acquired images was uniformly set to 640 pixels × 480 pixels.

#### 2.1.1 Image acquisition parameters and construction of complex scenarios

**Figure 2:**
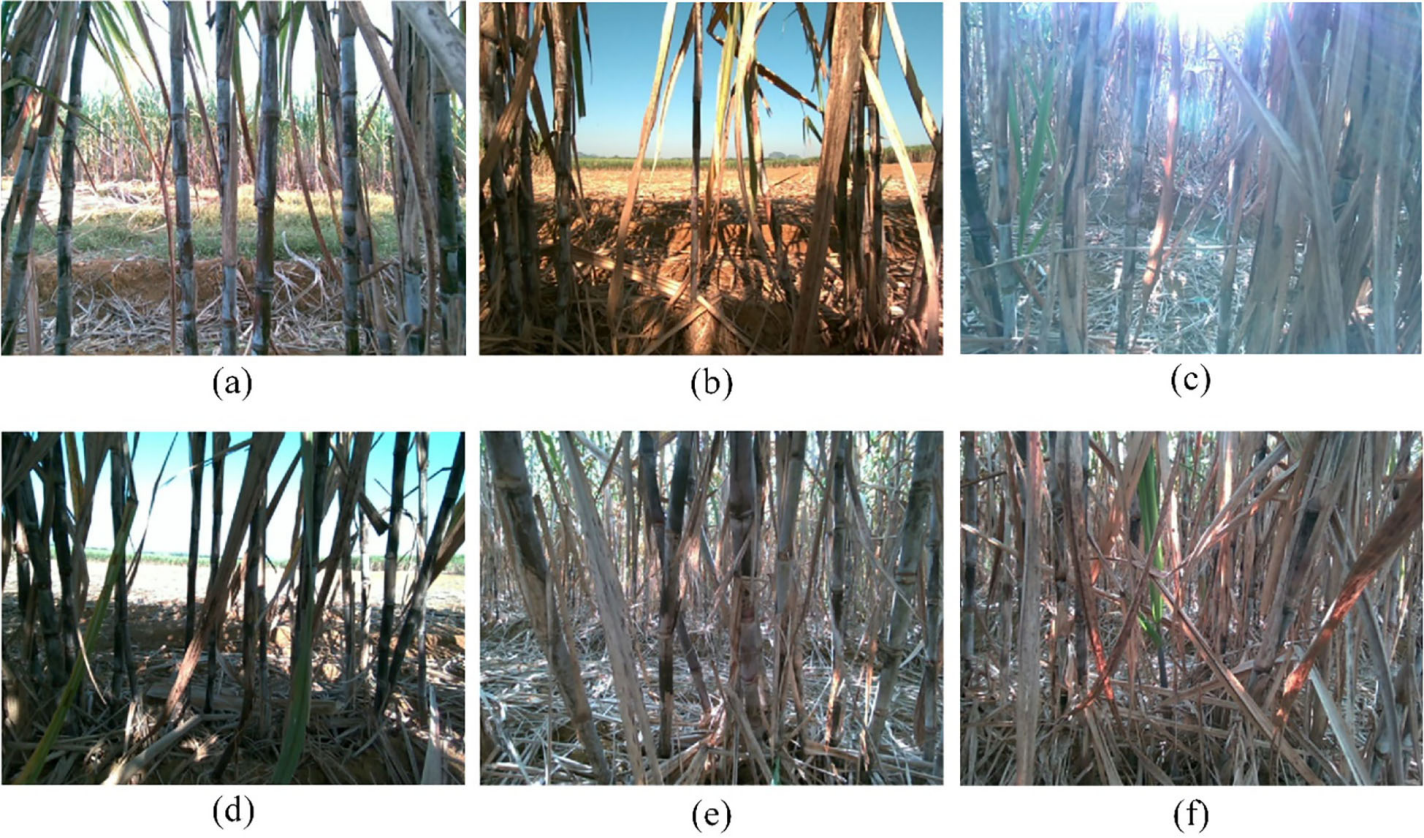
Multiscenario image samples of sugarcane in the field. **(a)** Ideal lighting scenario, **(b)** front-lit scenario, **(c)** backlit scenario, **(d)** backlit with local shadow scenario, **(e)** high-density distribution scenario, and **(f)** heavy dead leaf coverage scenario.

The sugarcane farmland environment has significant dynamic characteristics. If only a single data-driven model under an ideal scenario is used, the network will likely overfit the environment during the actual inference stage. Therefore, during the construction phase of this dataset, the proportion of “difficult samples” was deliberately increased. A total of 1–264 valid images of SSs in the field were ultimately selected, with the various scenarios shown in **Figure 2**. To meet the requirements for model training and the objective evaluation of model generalizability, a random sampling strategy was adopted, with the image set divided into training (1–011 images), validation (127 images), and testing (126 images) sets at an 8:1:1 ratio.

#### 2.1.2 Annotation rules for instance segmentation

**Figure 3:**
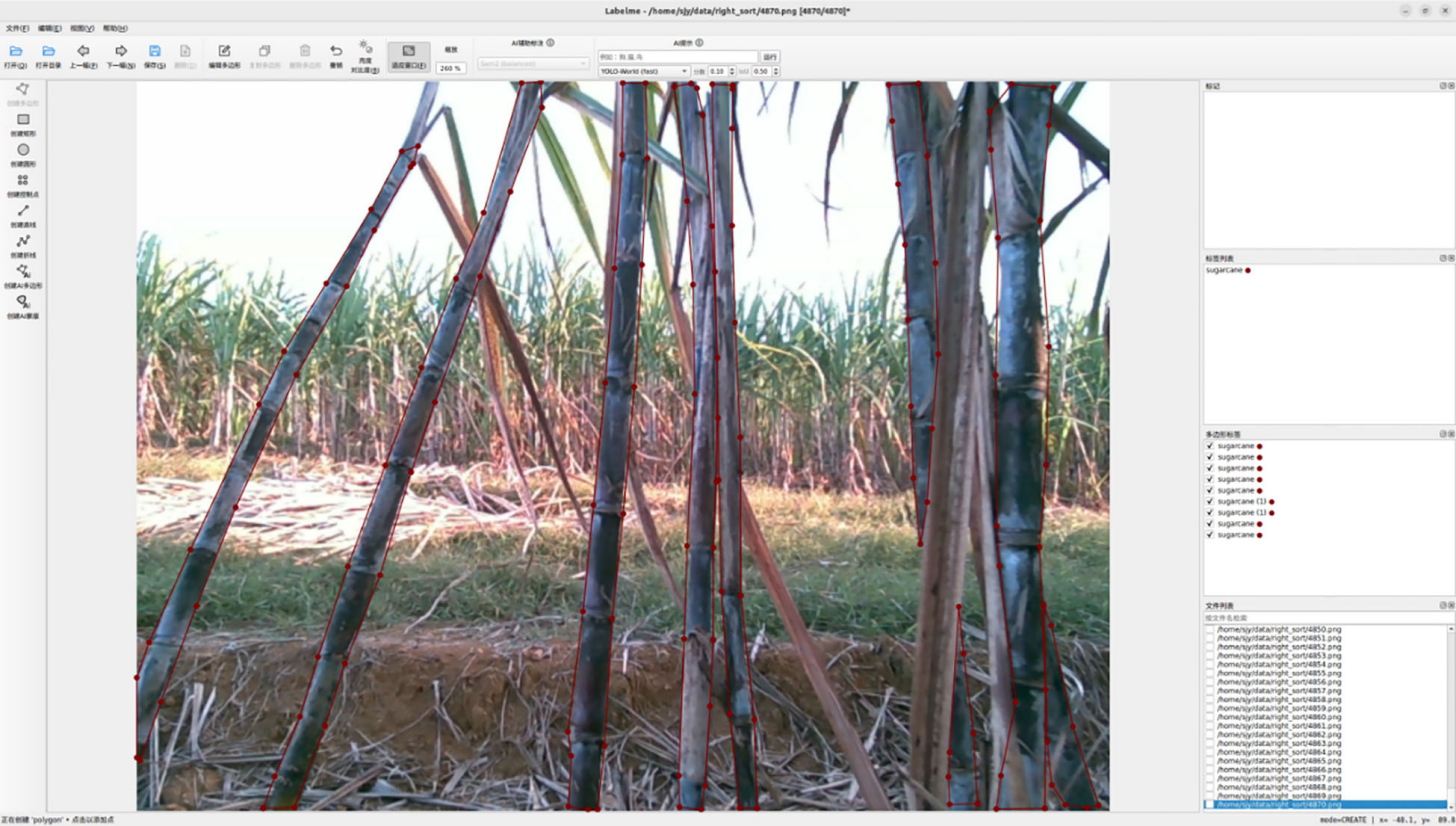
Fine-grained polygon annotation process for SS using Labelme.

After c the SS images in the field were collected, pixel-level instance annotation of the sugarcane image data was necessary. Traditional object detection methods typically use bounding boxes to localize targets. However, sugarcane has an elongated structure, and sugarcane is densely distributed with stems that easily occlude one another in the field. If bounding boxes are forced, they inevitably contain a large number of background weed pixels or pixels from adjacent SSs (**Figure 3**). Moreover, severe overlap among the SSs leads to high IoU values between adjacent bounding boxes. During the NMS stage of model inference, the system can easily mistake real SSs as redundant prediction boxes and erroneously delete them, resulting in missed detections.

To address the missed detection problem caused by the erroneous removal of redundant prediction boxes during the NMS stage in bounding-box-based object modelling, the polygon tool in Labelme (Russell et al., 2008) software was employed for pixel-level instance segmentation annotation, with the category uniformly labelled as “sugarcane”.

#### 2.1.3 Statistical analysis of the geometric features of the training set labels

**Figure 4:**
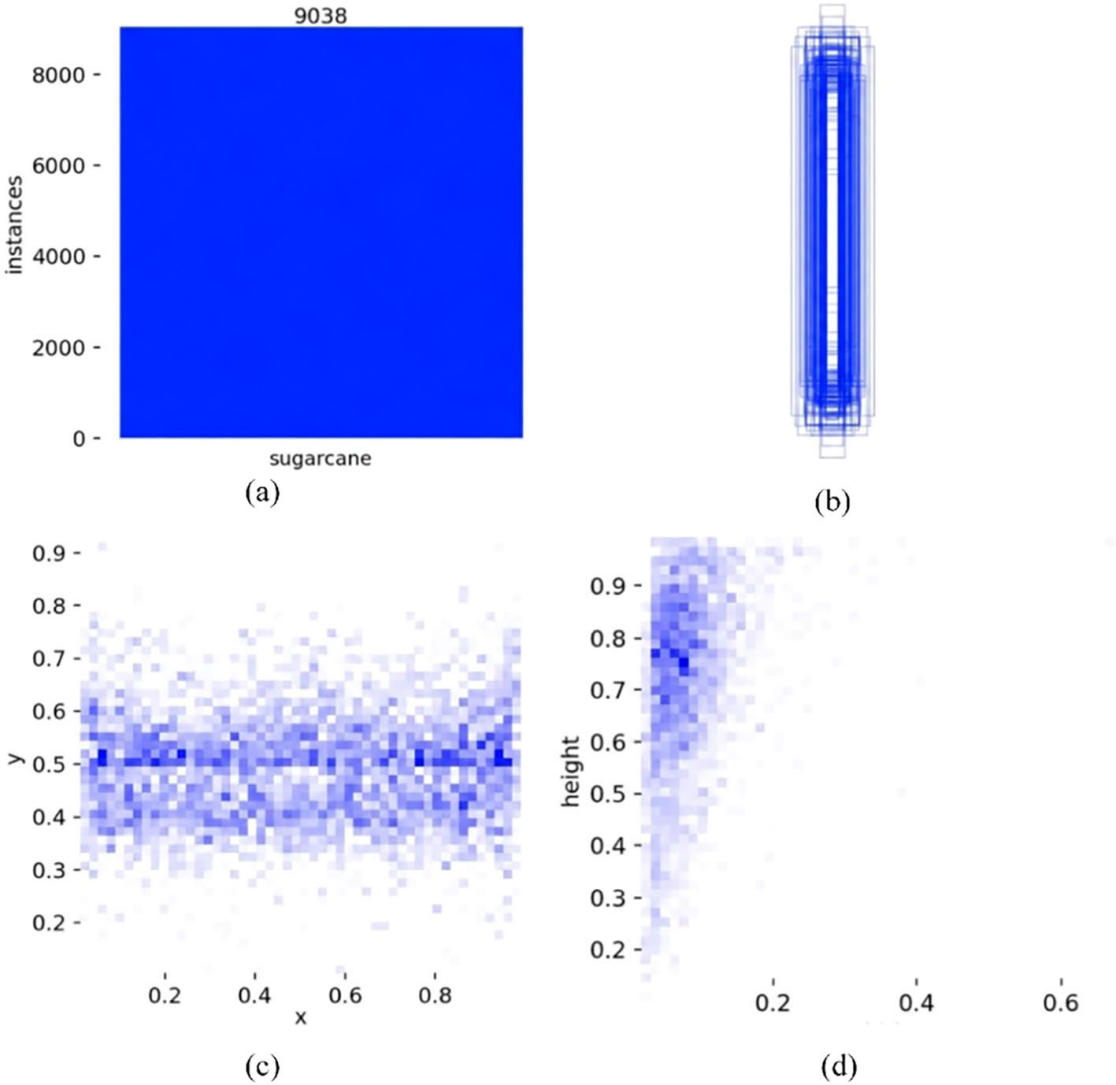
Statistical and geometric spatial distributions of the SS instance labels in the training set. **(a)** Histogram, **(b)** size projection plot, **(c)** center coordinate distribution plot, and **(d)** height scatter distribution plot.

After the format conversion was complete and the dataset was randomly divided into 8:1:1 splits, a global feature statistical analysis was performed on the training set containing 1–011 images using an analysis script. The resulting geometric morphology and spatial distribution are shown in **Figure 4**. This set of statistical data objectively reflects the physical spatial distribution patterns of the SS targets and provides a data-driven basis for the targeted improvements to the RT-DETR-seg model.

As shown in the histogram in **Figure 4a**, a total of 9–038 valid polygon instances of SSs were retained in the training set. The sufficient quantity and quality-checked valid samples effectively support the updating and convergence of the large number of parameters in the deep learning model. The size projection plot in **Figure 4b** and the width vs. height scatter plot in the lower right corner reveal that most SSs have relative widths between 5% and 20%, while the relative height is distributed in the range of 0.3 to 0.9. This finding indicates that the SSs exhibit very pronounced “tall and thin” characteristic in the images. If traditional square convolutional kernels (CKs) are used for feature extraction, substantial weeds and background noise on both sides of the target will be incorporated into the bounding boxes when the SS features are acquired. This provides a data-driven basis for targeted improvements to the RT-DETR-seg model. The center coordinate distribution plot (x vs. y) in **Figure 4c** shows that the center points of the sugarcane targets are relatively uniformly distributed in the horizontal direction (X-axis), but are highly concentrated in the vertical direction (Y-axis) within the core central region of the image, ranging from 0.4 to 0.6.

#### 2.1.4 Data augmentation preprocessing strategy

**Figure 5:**
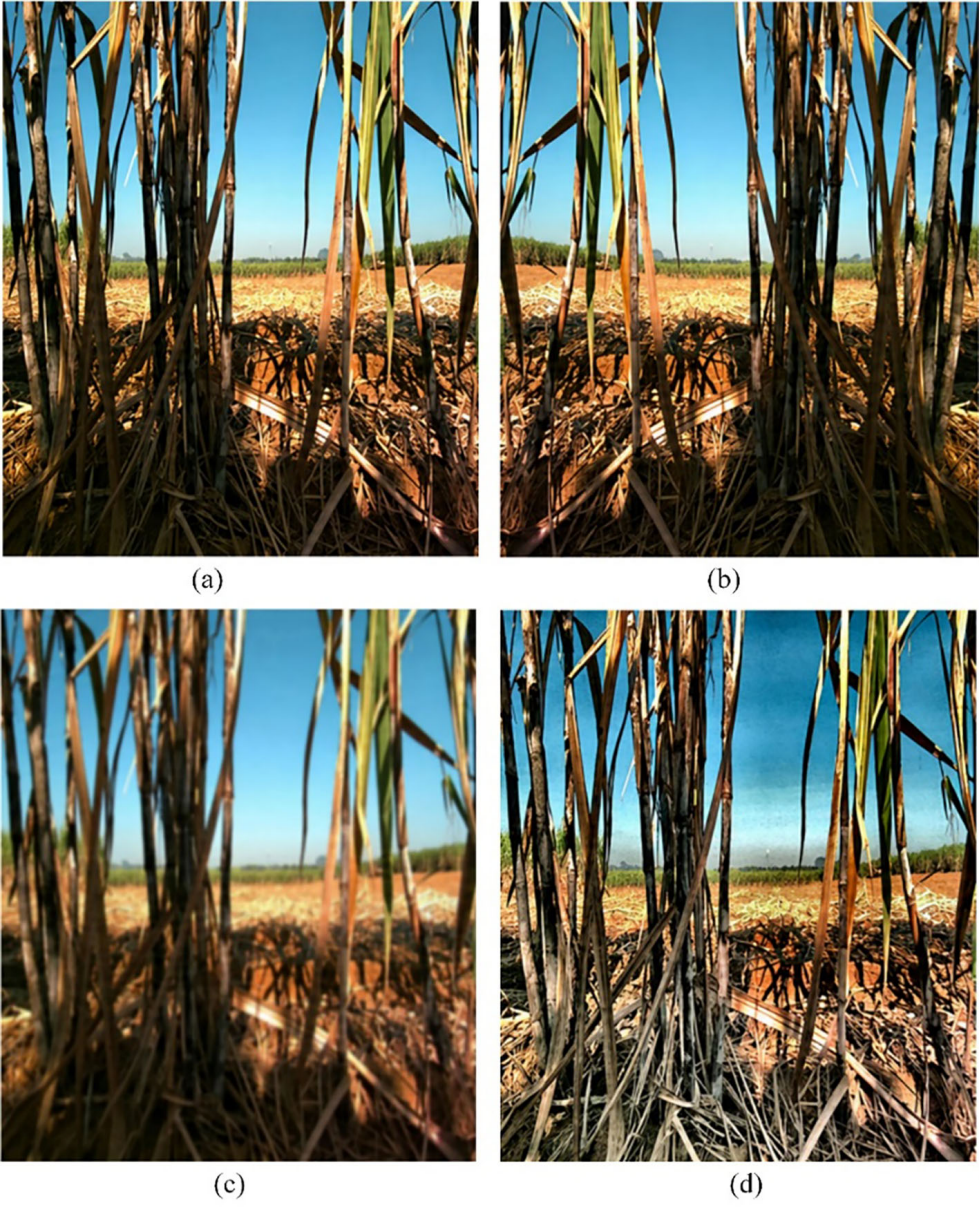
Comparison of offline data preprocessing effects: **(a)** original SS image; **(b)** horizontal flip; **(c)** Gaussian blur; and **(d)** CLAHE equalization.

Considering that the RT-DETR-seg model might overfit the data when the training set includes only 1–264 SS images, and to increase the adaptability of the model in complex sugarcane field environments, a strategy combining offline augmentation with online augmentation was adopted to address issues such as illumination variation and SS overlap (**Figure 5**). During the implementation of various spatial transformations, the algorithm synchronously maps image pixels and mask coordinates, thereby ensuring the accuracy of the supervision signals.

##### 2.1.4.1 Offline data augmentation of SS images

###### 2.1.4.1.1 Horizontal flip

Considering the physical characteristic of sugarcane, namely, that is consistently grows upward, only horizontal mirror flipping was employed during data augmentation. Assuming that the original image size is W × H and that the initial pixel coordinates are (x, y), the transformed coordinates (x’, y’) are computed according to the homogeneous coordinate matrix as shown in Equation 1:

(1) $$\left[\begin{array}{c} x^{\prime }\\ y^{\prime }\\ 1 \end{array}\right]=\left[\begin{array}{ccc} -1 & 0 & W-1\\ 0 & 1 & 0\\ 0 & 0 & 1 \end{array}\right]\left[\begin{array}{c} x\\ y\\ 1 \end{array}\right]$$

###### 2.1.4.1.2 Gaussian blur

This Gaussian blur operation aims to restore the blurring effect of the SS image during the collection of SS images, thereby reducing the model’s reliance on high-frequency textures and enabling the algorithm to acquire more robust geometric shape features.

###### 2.1.4.1.3 Contrast limited adaptive histogram equalization

To address the issues of backlighting and shadow interference in SS images collected in the field, the CLAHE algorithm was introduced to remap the probability density function of the local regions in the image. This method significantly restored the edge details of the SSs in the dark areas without introducing overexposure noise (Pal and Gupta, 2026).

##### 2.1.4.2 Online dynamic augmentation

In this study three online augmentation strategies were introduced through the Dataloader during model training:

###### 2.1.4.2.1 Random rotation

This approach aims to simulate the tilted posture of sugarcane caused by wind or natural lodging conditions. A rotation transformation matrix (Equation 2) was used to fine-tune the images and corresponding masks within θ ∈ [−15°, 15°] to improve the model’s ability to recognize nonvertical targets:

(2) $$\left[\begin{array}{c} x^{\prime }\\ y^{\prime } \end{array}\right]=\left[\begin{array}{cc} \cos\theta & -\sin\theta \\ \sin\theta & \cos\theta \end{array}\right]\left[\begin{array}{c} x\\ y \end{array}\right]$$

###### 2.1.4.2.2 Mosaic augmentation

**Figure 6:**
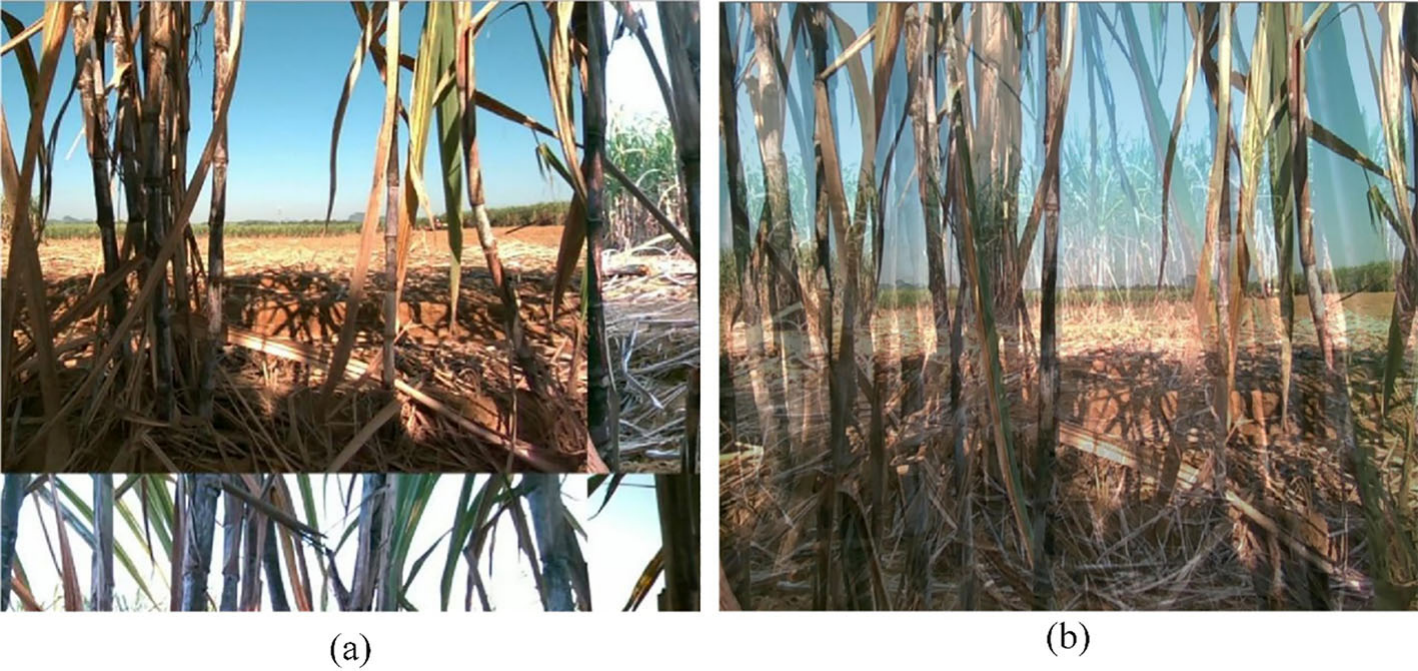
Illustration of online data augmentation effects. **(a)** Mosaic augmentation; and **(b)** mix-up augmentation.

Four images were randomly sampled from the dataset, scaled, cropped, and then stitched together. This strategy alters the relative spatial proportions of the targets, artificially increasing the instance density within a single frame (**Figure 6a**), thereby increasing the model’s detection sensitivity to dense small targets (Bochkovskiy et al., 2020).

###### 2.1.4.2.3 Mix-up augmentation

Two random samples and their mask labels were linearly weighted and fused (**Figure 6b**). Assuming that the two images and their labels are (*x*_i_, *y*_i_) and ( $\tilde{x},\;\tilde{y}$), the synthesized virtual sample follows Equation 3:

(3) $$\{\begin{array}{l} \tilde{x}=\lambda x_{i}+\left(1-\lambda \right)x_{j}\\ \tilde{y}=\lambda y_{i}+\left(1-\lambda \right)y_{j} \end{array}$$

where λ represents a weight parameter following a beta distribution. The pixel-level virtual overlap effect constructed by mix-up operation forces the network to learn to separate individual instances from overlapping pixels, thereby effectively mitigating the missed detection problem in stem-crossing scenarios (Zhang H. et al., 2018).

### 2.2 Methods

#### 2.2.1 Overall design of RT-DETR-seg

**Figure 7:**
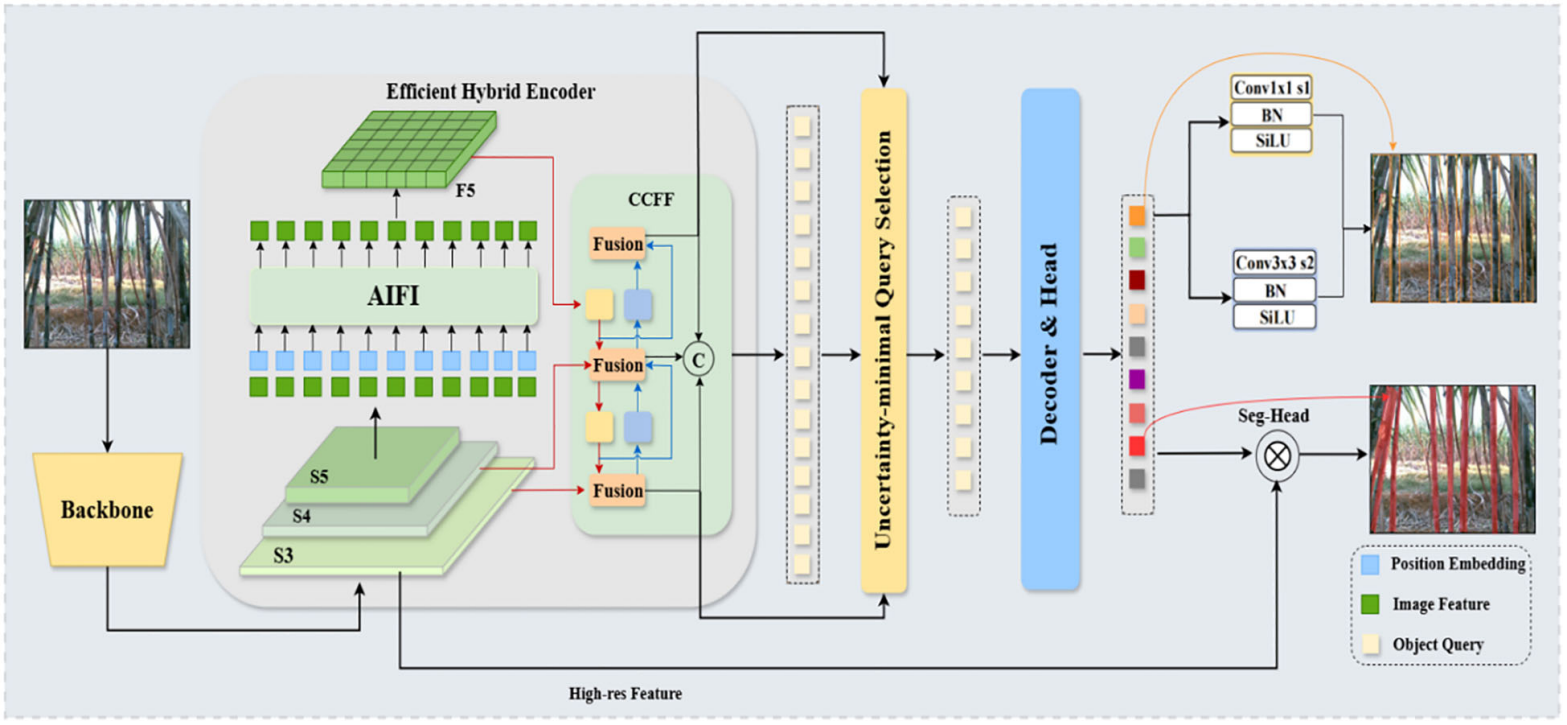
Baseline network architecture of the sugarcane stem instance segmentation network based on RT-DETR-seg.

The emergence of the DETR network series has changed the previous detection paradigm, which relied on dense anchor boxes, and transformed object detection into a set prediction problem based on bipartite matching. RT-DETR-seg shares this end-to-end design. Furthermore, to address the excessive computational cost when transformers process high-resolution image features, an efficient hybrid encoding mechanism is incorporated. The overall data processing flow of the model is shown in **Figure 7**. Image data sequentially pass through the backbone feature extraction network, the hybrid encoder, and the transformer decoder, finally, both the bounding boxes and pixel-level segmentation masks of the targets are output.

##### 2.2.1.1 Backbone feature extraction network

The visual feature extraction backbone of RT-DETR-seg relies on a high-performance CNN, typically ResNet or the HGNetv2 architecture. When a field sugarcane image of size H × W × 3 is input to the network, the backbone performs bottom-up multiscale feature extraction via multiple alternately stacked residual modules and downsampling layers.

At the shallow network stage, the CKs are primarily responsible for capturing local high-frequency information, such as the physical edges, surface texture, and color gradients of the SSs. As the network depth increases and the receptive field progressively expands, the deep layers gradually transition from low-level geometric features to high-level abstract semantic features. Finally, the backbone outputs three feature maps with different resolution (downsampled by factors of 8, 16, and 32), providing hierarchical spatial information for subsequent cross-scale feature interaction.

##### 2.2.1.2 Hybrid encoder

To overcome the computational bottleneck of standard vision transformers, where the computational cost is a quadratic when high-resolution images are being processed, RT-DETR-seg includes an innovatively designed hybrid encoder. This module is composed of an attention-based intrascale feature interaction (AIFI) module and a cross-scale feature fusion module (CCFM) that work in synergy.

The AIFI module focuses on processing the highest-level abstract semantic features output by the backbone, which is the feature map downsampled by a factor of 32. By introducing a self-attention mechanism, the model establishes long-range global dependencies on the low-resolution, high-semantic-dimension feature map (Vaswani et al., 2017). The core attention operation is shown in Equation 4:

(4) $$Attention\left(Q,K,V\right)=softmax\left(\frac{QK^{T}}{\sqrt{d_{k}}}\right)V$$

where Q (Query), K (Key), and V (Value) are all obtained by linearly transforming the deep feature tensor, and d_k_ is the channel dimension scaling factor. Through this mechanism, the network can capture the macroscopic spatial distribution patterns of sugarcane targets on a global scale. The deep features enhanced by the AIFI module are subsequently sent to the CCFM. The CCFM employs a bidirectional fusion strategy involving top-down and bottom-up methods, similar to the feature pyramid network (FPN), to seamlessly combine the rich semantic information from deep layers with the precise local details from shallow layers across different scales. This fusion mechanism ensures that the feature tensor input to the decoder not only has a global perspective but also does not lose the fine boundary features required for the segmentation task.

##### 2.2.1.3 Transformer decoder and segmentation head (Seg-Head)

The most groundbreaking aspect of the RT-DETR-seg architecture is that its decoder completely eliminates the reliance of traditional models on anchor boxes and postprocessing through NMS. The decoder initializes a set of fixed learnable object queries, and in the cross-attention module, the query vectors are used to actively “probe” and aggregate the target features within the global feature map, thereby gradually refining the position and category attributes of the sugarcane instances.

During the gradient update stage of model training, RT-DETR-seg employs the Hungarian algorithm to perform optimal bipartite matching between the predicted set and the true ground-truth (GT) labels. The minimization logic of the bipartite matching cost function is as shown in Equation 5:

(5) $$\hat{\sigma }=\arg\underset{\sigma \in S_{N}}{\min}\sum_{i=1}^{N}{ℒ}_{match}\;\left(y_{i},{\hat{y}}_{\sigma \left(i\right)}\right)$$

where y_i_ represents the true label of the i-th real sugarcane instance, and ${\hat{y}}_{\sigma \left(\text{i}\right)}$ is the predicted output of the network under a certain permutation and combination. This end-to-end collective prediction mechanism eliminates the theoretical risks of false deletion and omission that may occur in dense overlapping scenarios at the base algorithmic level. For IS tasks, clear boundary separation is crucial. Therefore, the model includes a dedicated Seg-Head at the decoder end, as shown in **Figure 7**. The Seg-Head component the optimized query embedding from the decoder and performs a pixelwise dot product operation with the high-resolution shallow feature map output by the hybrid encoder. Through this multidimensional mapping mechanism, the network re-projects the abstract target semantic features back to the 2D image space, ultimately generating a binary mask representing each individual SS.

Identifying the performance bottlenecks of the baseline model in the processing of unstructured field targets is a prerequisite for subsequent algorithm improvement efforts. Combined with the above statistical analysis of sugarcane spatial characteristics, the limitations of the RT-DETR-seg model in practical applications are reflected primarily in the following three aspects.

First, there is a physical mismatch between the CK shape and the target geometry. The data in **Figure 4** show that SSs exhibit extreme “tall and thin” characteristics in the images, with a relative width typically only 5% to 20%. However, the original backbone network still uses 1:1 square CKs. When such square windows act on elongated targets, both sides of the window inevitably incorporate substantial background noise such as soil and weeds, thereby interfering with the model’s ability to accurately capture the main features of the sugarcane.

Second, the problem of individual feature fragmentation caused by physical occlusions is significant. In the mid-to-late growth stages, SSs are commonly truncated owing to occlusions from broad leaves. Owing to the lack of effective constraints on the “spatial coherence” of slender targets in the original model, the decoder is prone to mistakenly identifying the same plant as multiple independent individuals when dealing with such discontinuous features. This ultimately leads to duplicate counting errors.

Third, the pixel-level loss function has insufficient constraining power under complex lighting conditions. The segmentation branch of the baseline model relies mainly on the cross-entropy or Dice loss for optimization. These functions account for only the pixel-level classification accuracy. Since the loss function fails to fully utilize the geometric prior knowledge of sugarcane, which is characterized by a large aspect ratio and relatively smooth edges, the model has difficulty generating accurate and smooth masks in areas with intense light changes.

On the basis of the morphology of sugarcane, to address the issues of field shading and light interference, three improvements were made to the RT-DETR-seg baseline model. First, asymmetric strip convolutions (ASCs) were introduced into the main network to enhance longitudinal features and filter out weed noise. Second, the coordinate attention (CA) mechanism was embedded in the hybrid encoder to reassociate the truncated stems using long-range spatial (LRS) connections. Third, the MGR loss function was designed to improve the boundary segmentation quality under complex lighting conditions through an aspect ratio penalty and gradient smoothing constraints.

#### 2.2.2 Feature extraction network incorporating ASCs

Given the limitations of the original baseline model in complex agricultural field scenarios, the structure of the backbone feature extraction network is improved from the perspective of matching the feature extraction window with the target morphology.

Existing vision backbone networks such as ResNet generally rely on square regions such as 3×3 or 5×5 regions for local feature aggregation. However, according to the spatial distribution statistics of the annotated data in **Figure 4**, SSs in the field exhibit extremely pronounced slender characteristics, with the relative pixel width of a single plant typically being less than 20% of the total image width. In such environments, when traditional square CKs are used to extract features, the horizontal windows inevitably incorporate many background pixels such as adjacent soil, weeds, and dead leaves. To eliminate this mismatch between the extraction window and the target geometry, an ASC module was designed and introduced.

In the construction of this network module, on the basis of the prior statistics of the SS morphology, the span parameter *N* of the strip convolution kernel was set to 9. The ASC module adopts a lightweight dual-path parallel computation mechanism, and its forwards feature flow proceeds as follows.

First, the input feature map passes through a 1×1 point convolution layer to compress the channel dimension and integrate cross-channel contextual information, thereby reducing the computational load of subsequent parallel branches. This intermediate feature is subsequently fed into two asymmetric branches: a 9×1 vertical convolution branch focusing on vertical features and a 1×9 horizontal convolution branch that captures horizontal features. To ensure precise spatial alignment of the output feature resolutions across the parallel streams, zero padding with size 4 is employed in both branches. Finally, the output tensors from both branches are combined using elementwise addition, and the resulting enhanced feature is mapped through a nonlinear activation function. The mathematical expression of the above forwards mapping process is as follows in Equations 6, 7:

(6) $$X_{mid}=\sigma \left(B_{mid}\left(X*W_{1\times 1}\right)\right)$$

(7) $$Y=\sigma \left(B_{v}\left(X_{mid}*W_{9\times 1}\right)+B_{h}\left(X_{mid}*W_{1\times 9}\right)\right)$$

where * denotes the 2D convolution operation; and W_1×1_, W_9×1_, and W_1×9_ represent the CK weight matrices for the corresponding branches. B represents two-dimensional batch normalization, which is used to prevent the gradient dispersion phenomenon in deep networks. *σ* represents the SiLU activation function. This function is chosen to align strictly with the underlying nonlinear mapping characteristics of the benchmark RT-DETR-seg model.

**Figure 8:**
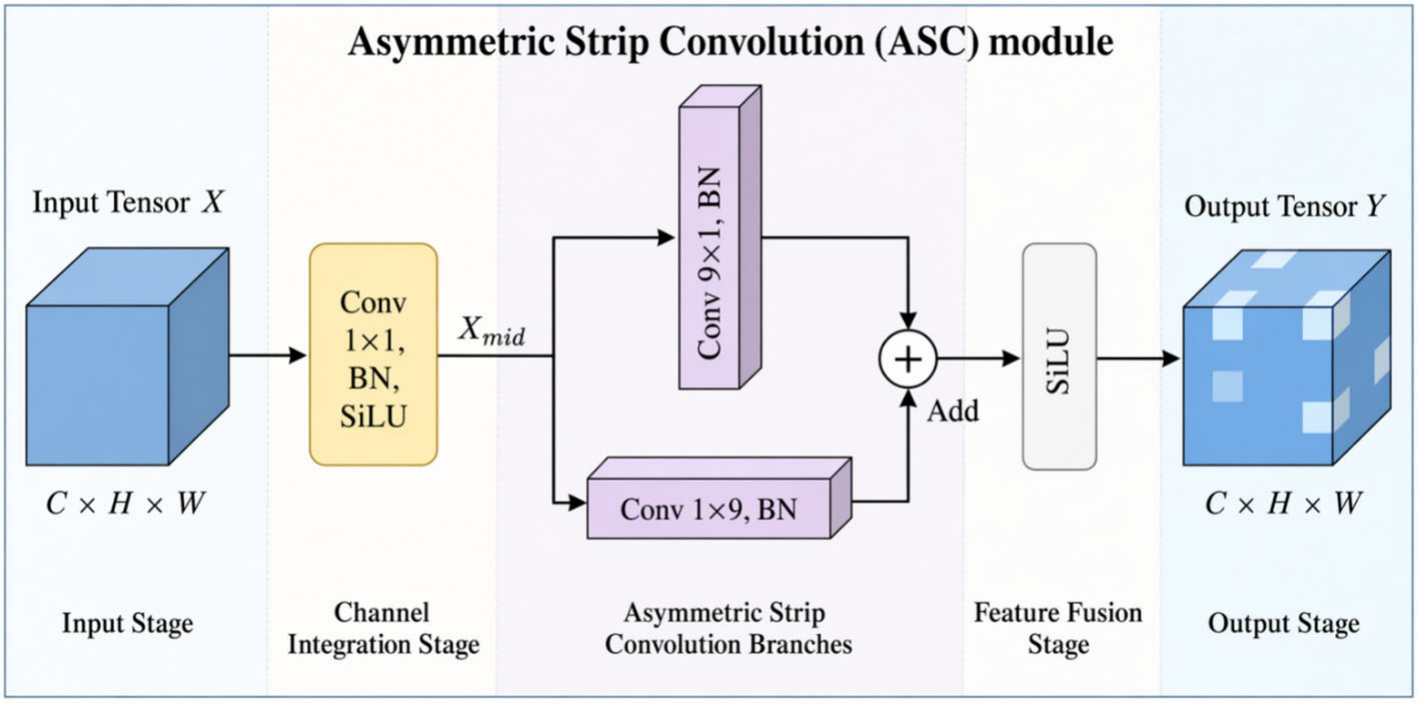
Internal structure diagram of the ASC module.

Furthermore, from the perspective of computational complexity, although large 9×9 - square convolution kernels can cover elongated targets, the use of such large kernels introduces many parameters and floating-point operations (FLOPs). The asymmetric decoupling design adopted in this paper (decomposing the two-dimensional space into 1×9 and 9×1 branches) significantly reduces the number of parameters from O(N^2^) to O(2N) while obtaining the same one-way spatial span. This lightweight module design enhances the ability of the model to recognize occlusionsy while meeting the real-time requirements of the inspection platform. In terms of specific deployment, the ASC module is applied only to the P5 feature output end of the main network (**Figure 8**) and serves, as a transition transformation mechanism before the feature enters the hybrid encoder. This level has the largest receptive field and the lowest spatial resolution; thus, the overall elongated structure of the SSs can be effectively captured with minimal increases in the computational cost.

#### 2.2.3 Long-range dependency modelling with the CA mechanism

Although the ASC module improves feature extraction for elongated targets, the local nature of the convolution operations still imposes certain limitations on its performance under extreme environmental conditions. In particular, when the SSs are extensively covered by dead leaves or when stems cross each other, the continuity of the target often cannot be guaranteed. Therefore, the CA mechanism is introduced and adopted in this paper (Hou et al., 2021). By embedding precise positional information, the model’s ability to capture LRS dependencies is enhanced, allowing semantic associations that were lost because of occlusions to be restored from a global perspective.

##### 2.2.3.1 Principle of coordinate attention position encoding

**Figure 9:**
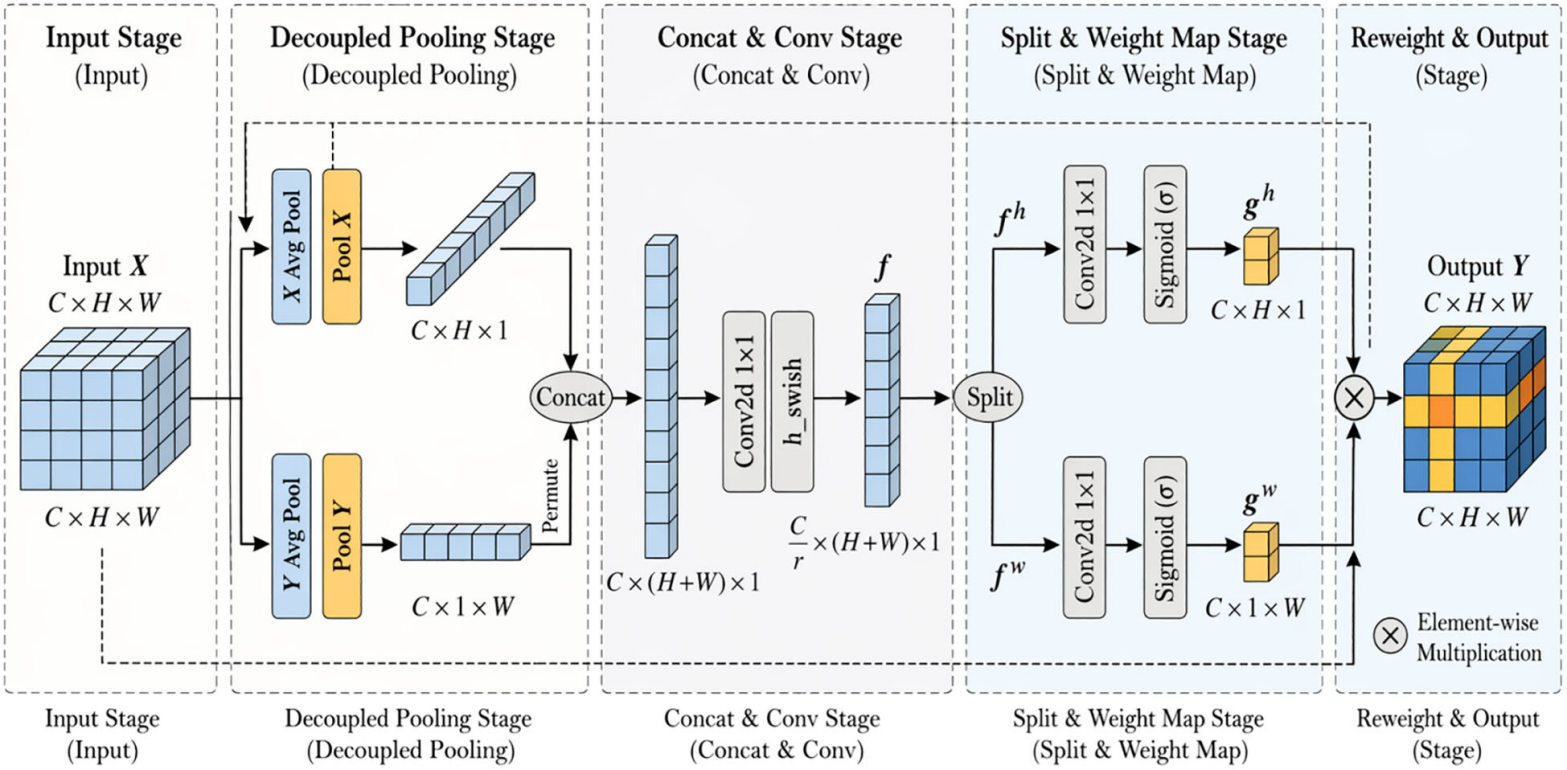
Illustration of the coordinate attention position encoding and feature mapping process.

In visual tasks, traditional attention mechanisms, such as the squeeze and excitation module (Hu et al., 2018), typically compress two-dimensional feature maps into one-dimensional scalars via global average pooling strategies. Although this approach reduces the computational overhead, it also leads to the loss of some important spatial information. In contrast, the CA mechanism adopts a more refined approach by decomposing channel attention into encoding along two directions: horizontal (X-axis) and vertical (Y-axis). The structure and feature mapping process of the CA module are illustrated in **Figure 9**.

During the decoupled pooling stage, given an input feature tensor $\text{X}\in {\mathbb{R}}^{\text{C}\times \text{H}\times \text{W}}$, the CA module first aggregates information in the spatial coordinate system using adaptive average pooling kernels of sizes (H, 1) and (1, W) (Hu et al., 2018). For channel *c*, the feature output at height h along the horizontal direction can be expressed as follows in Equation 8:

(8) $${\text{z}}_{\text{c}}^{\text{h}}\left(\text{h}\right)=\frac{1}{\text{W}}\sum_{0\leq \text{i}<\text{W}}{\text{x}}_{\text{c}}\left(\text{h},\text{i}\right)$$

A feature output with width *w* in the vertical direction can be represented as follows in Equation 9:

(9) $$z_{c}^{w}\left(w\right)=\frac{1}{H}\sum_{0\leq j<H}x_{c}\left(j,w\right)$$

After the directional pooling operations, the network extracts two directional vectors that capture the horizontal and vertical distribution patterns of the target. Next, in the Concat & Conv Stage, the feature maps from these two directions, *z*^h^ and *z*^w^, are concatenated along the spatial dimensions and fed into a shared 1×1 convolutional layer for dimensionality reduction. The resulting feature flow then passes through a smooth *h-swish* activation function *δ* to generate a nonlinearly mapped intermediate transitional feature tensor *f* (Hou et al., 2021) and the calculation process is as shown in Equation 10:

(10) $$\;f=\Phi \left(ℬ\left(Conv_{1\times 1}\left(\left[z^{h},z^{w}\right]\right)\right)\right)$$

where [·,·] denotes concatenation and ℬ represents batch normalization. After the nonlinear mapping, the tensor *f* is split along the spatial dimensions into two parts: the horizontal component $f^{h}\in {\mathbb{R}}^{\frac{C}{r}\times H\times 1}$ and the vertical component $f^{w}\in {\mathbb{R}}^{\frac{C}{r}\times 1\times W}$. These components are then individually processed by their respective 1×1 convolutions followed by *sigmoid* activation functions σ, producing corresponding attention weight vectors *g*^h^ and *g*^w^ (Equations 11, 12):

(11) $$g^{h}=\sigma \left(Conv_{h}\left(f^{h}\right)\right)$$

(12) $$g^{w}=\sigma \left(Conv_{w}\left(f^{w}\right)\right)$$

Finally, in the Reweight & Output Stage, the CA module multiplies the coordinate-aware weights with the original input feature map via an elementwise operation, generating the enhanced feature *y_c_.* In this way, the output features retain the original information while incorporating positional cues:

(13) $$y_{c}\left(i,j\right)=x_{c}\left(i,j\right)\times g_{c}^{h}\left(i\right)\times g_{c}^{w}\left(j\right)$$

##### 2.2.3.2 Cross scale embedding strategy and location logic

**Figure 10:**
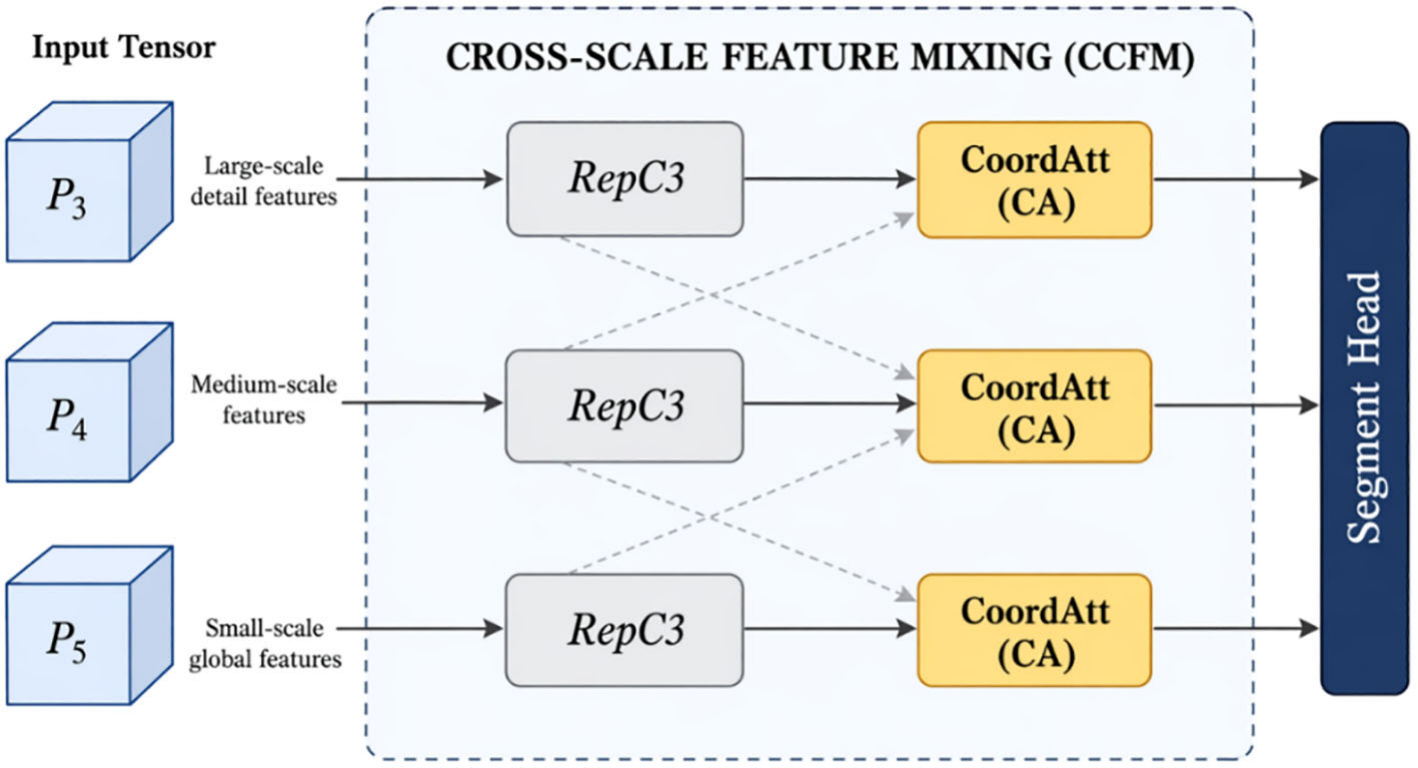
Embedding position information in the CA module in the improved RT-DETR hybrid encoder.

On the basis of the topology of the RT-DETR-seg network, a multiscale end-embedding strategy is adopted in this paper. The CA module is precisely embedded into the CCFM of the RT-DETR hybrid encoder. In accordance with the hierarchical evolution logic of the model architecture (**Figure 10**), the CA module is deployed at the end of three feature scales, P3, P4, and P5, i.e., the final stage before the feature stream converges in the Seg-Head component. This “multiscale collaborative embedding” design follows the following logical reasoning:

Shallow-layer detail correction (P3 branch): At the high-resolution feature level, the response of edge coordinates is enhanced, interclass interference from background weeds is suppressed, and the Seg-Head component is assists in generating smooth masks that conform to the physical boundaries of the SSs.

Mid-layer instance decoupling (P4 branch): To address mask adhesion issues caused by the dense distribution of the SSs, a horizontal attention mechanism is used to enhance the model’s perception of individual physical gaps, thereby improving instance discrimination.

Deep-layer semantic reconstruction (P5 branch): At the low-resolution feature level, the model can observe a relatively large range of image information. Introducing coordinate encoding at this level helps establish spatial associations for occluded or truncated SS targets.

#### 2.2.4 Loss function improvement incorporating geometric constraints and photometric robustness

##### 2.2.4.1 Limitations of the mask loss function

In typical instance segmentation models, such as the benchmark RT-DETR-seg model, the segmentation head usually employs the BCE loss and dice loss to jointly apply constraints for mask prediction. These losses are optimized mainly at the pixel level, with a focus on whether each pixel is correctly classified. Thus, the model pays more attention to the single-pixel prediction results but does not consider the shape or structural continuity of the target objects.

On the basis of the above statistical analysis of sugarcane environments in the field, when only pixel-level constraints are used, the stability of the model in complex scenarios is insufficient. If the local area is affected by strong backlighting, tree crown shadows or other light and shadow interference, the mask boundary is prone to irregular fluctuations. If no geometric shape constraints are applied and only pixel-level losses are used for training, the model usually faces the following problems:

Morphological distortion problem under light and shadow interference conditions: The geometric statistical results in “Section 2.1.3 (“Statistical Analysis of Geometric Features of the Training Set Labels”) reveal, that individual sugarcane targets in the image mostly exhibit a “tall and thin” characteristics, and the relative width of the sugarcane is usually concentrated between 5% and 20% of the image width. Traditional loss function do not explicitly constrain this geometric feature; thus, in areas with strong light and shadow changes, the model is prone to semantic confusion. For example, some similar colors may be misjudged as the sugarcane body, resulting in the generated mask expanding irregularly towards the background area.

**Figure 11:**
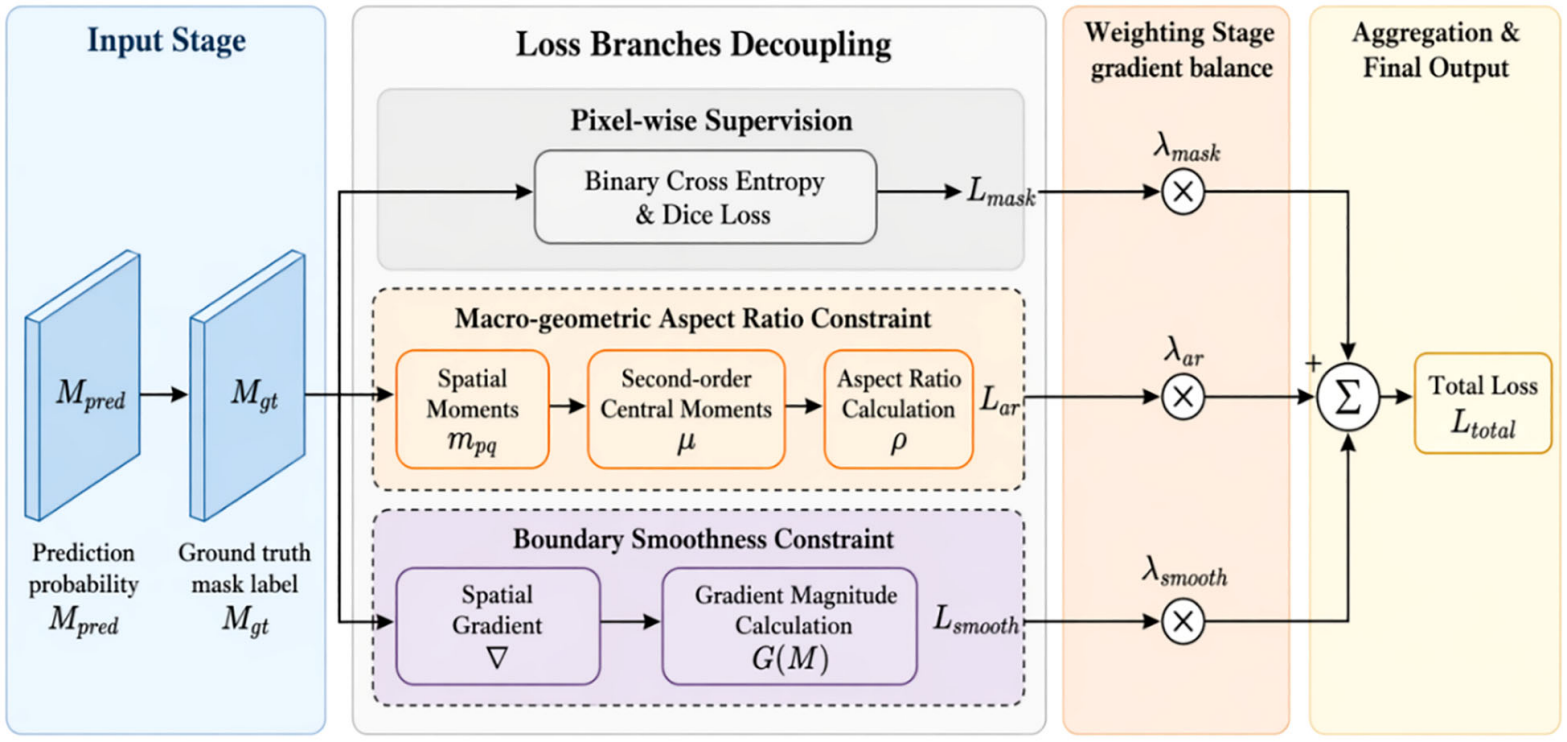
Topology of the joint loss function incorporating geometric constraints.

On the basis of the above analysis, in this paper, the loss function of the benchmark model is improved, and image space moments and gradient field constraints are introduced (**Figure 11**), enabling the geometric shape features of the sugarcane to be considered during model training. This strategy can reduce the interference of complex field environments on mask prediction and increase the stability of the segmentation results.

##### 2.2.4.2 Construction and derivation of the aspect ratio penalty term and the edge smoothing constraint

To make the predicted masks more consistent with the actual morphology of sugarcane, t a moment gradient regularized (MGR) loss, which combines spatial moment constraints and gradient field constraints, is designed in this paper. This loss introduces geometric morphology constraints and edge smoothness constraints during training, which aim to mitigate the impact of extreme lighting conditions and shadows on the predicted masks and thus increase the stability of the segmentation results.

###### 2.2.4.2.1 Aspect ratio penalty based on second-order central moments (L_ar_)

SSs typically exhibit elongated morphologies; therefore, the vertical distribution of their masks should be significantly greater than the horizontal distribution. To describe this morphological feature, spatial image moments are used to represent the spatial distribution of the predicted mask M_pred_. Assuming that M(x, y) is the continuous probability map output by Seg-Head and processed by the sigmoid activation function (soft mask, where M(x, y) ∈ (0, 1), the (p + q)-order spatial moment is defined as follows in Equation 14:

(14) $${\text{m}}_{\text{pq}}=\sum_{\text{x}=1}^{\text{W}}\sum_{\text{y}=1}^{\text{H}}{\text{x}}^{\text{p}}{\text{y}}^{\text{q}}\text{M}(\text{x},\text{y})$$

After deriving the centroid coordinates (x_c_, y_c_) are derived from the zero-order moment m_00_ (representing the soft area) and the first-order moments, the spatial distribution variances of the mask along the X-axis and Y-axis are defined as follows in Equations 15, 16:

(15) $$\text{Var}(\text{X})=\frac{\sum {\left(\text{x}-{\text{x}}_{\text{c}}\right)}^{2}\text{M}(\text{x},\text{y})}{{\text{m}}_{00}}$$

(16) $$Var\left(Y\right)=\frac{\sum {\left(y-y_{c}\right)}^{2}M\left(x,y\right)}{m_{00}}$$

Referring to the geometric feature statistics of the dataset shown in **Figure 4**, the mask aspect ratio characteristic ρ is defined as follows in Equation 17:

(17) $$\rho =\frac{\mathrm{Var}(X)}{\mathrm{Var}(Y)+\epsilon }$$

where ϵ is a very small constant to prevent division by zero. The aspect ratio penalty *L*_ar_ is constructed as follows in Equation 18:

(18) $$L_{\mathrm{ar}}=\frac{1}{N_{\mathrm{ins}}}\sum_{i=1}^{N_{\mathrm{ins}}}{\left(\rho_{\mathrm{pred}}^{\left(i\right)}-\rho_{\mathrm{gt}}^{\left(i\right)}\right)}^{2}$$

Through the gradient backpropagation mechanism, this constraint forces the mask to maintain an elongated morphology, effectively addressing the lateral expansion phenomenon caused by lighting and shadow interference.

###### 2.2.4.2.2 Perimeter consistency constraint based on the gradient field (*L*_smooth_)

To address the jaggedness problem caused by blurry edges, the spatial gradient operator ∇ is introduced. Considering that the surface of the SSs is relatively smooth, the mask edges should not exhibit excessive abrupt changes. Therefore, the spatial integral of the mask gradient should remain within a reasonable range. On this basis, the gradient magnitude map G(M) is defined as follows in Equation 19:

(19) $$\text{G}(\text{M})(\text{x},\text{y})=\sqrt{{\left(\frac{\partial \text{M}}{\partial \text{x}}\right)}^{2}+{\left(\frac{\partial \text{M}}{\partial \text{y}}\right)}^{2}+\epsilon }$$

The edge smoothness constraint *L*_smooth_ is defined as the mean squared error between the gradient magnitude maps of the predicted mask and the GT mask within the valid edge region Equation 20:

(20) $${\text{L}}_{\text{smooth}}=\frac{1}{{\text{N}}_{\text{edge}}+\epsilon }\sum_{\text{x}=1}^{\text{W}}\sum_{\text{y}=1}^{\text{H}}{\left(\text{G}\left({\text{M}}_{\text{pred}}\right)\left(\text{x},\text{y}\right)-\text{G}\left({\text{M}}_{\text{gt}},\text{y}\right)\right)}^{2}$$

where ϵ is a very small constant to prevent division by zero, and *N*_edge_ represents the total number of valid edge pixels of the GT mask (corresponding to the set of pixels where G(M_gt_) > 0). Considering that the distribution of the sugarcane target edges is extremely sparse across the image, directly using the full image area (W × H) for normalization would dilute the contribution of this loss term to a very low level. Therefore, the number of valid edge pixels *N*_edge_ is used for local normalization, forcing the network to focus precisely on the smoothness of the target contours while ensuring that its magnitude matches that of the pixel-level classification loss.

Furthermore, when the gradient magnitude map is computed, the spatial partial derivatives in Equation 13 can be implemented via finite-difference convolutions, such as horizontal and vertical edge extraction operators. These operators act on the continuous soft mask tensor, avoiding the nondifferentiability problem caused by step functions and ensuring normal backpropagation of the edge smoothness constraint gradient. During training, this constraint limits mask edge fluctuations in regions with abrupt lighting and shadow changes.

###### 2.2.4.2.3 Composite loss function design and multitask gradient balancing

Finally, the improved total loss function *L*_total_ consists of the detection error, mask classification error, and the proposed MGR loss. Their mathematical expressions are shown in Equations 21, 22:

(21) $$L_{MGR}=\lambda_{ar}L_{ar}+\lambda_{smooth}L_{smooth}$$

(22) $$L_{total}=L_{\text{det}}+{\text{λ}}_{\text{mask}}{\text{L}}_{\text{Dice+BCE}}+L_{MGR}$$

As shown in **Figure 10**, according to the joint supervision logic proposed in this study both the predicted mask and the GT mask are input into three parallel supervision branches: the global branch is responsible for pixel-level localization; the aspect ratio penalty branch constrains the morphological distribution on the basis of spatial moment theory; and the edge smoothness branch uses consistency of the gradient field to suppress edge jaggedness caused by lighting and shadow noise. These three branches, along with the detection loss, are weighted and summed to form the final joint loss function *L*_total_.

In multitask learning, balancing the gradient updates of different constraint terms is crucial. Conventional methods often rely on heuristic dynamic weighting or grid search strategies to bridge the initial dimensional differences between geometric penalties (second-order moments, gradient fields) and the classification loss (probability distribution), which can easily introduce artificial bias. To this end, a local normalization and ratio mapping strategy is adopted in the loss function. The output of the Seg-Head component is uniformly activated by a sigmoid function to generate a continuous probability map with a range of (0, 1). On the basis of the strict boundedness of the input tensor and the preceding local normalization procedure, the difference in magnitude between the derived geometric penalties and the classification loss at the beginning of training is significantly reduced. On the basis of this numerical alignment design, a heuristic minimalist equal-weight configuration (i.e., setting λ_mask_ = 1.0) is adopted, with the internal BCE and Dice weights both set to 0.5 and the geometric penalty weights set to λ_ar_ = 1.0 and λ_smooth_ = 1.0, without the need to introduce complex external bias constraints. With the AdamW optimizer, the network achieves a stable balance between microscopic pixel classification and macroscopic morphological constraints while eliminating the need for complex hyperparameter tuning.

#### 2.2.5 Hardware and software environment configuration

The model was trained and tested a Ubuntu 22.04 operating system. To manage dependency packages and avoid environmental conflicts, Conda was used to create an independent Python virtual environment. Code writing and debugging were primarily performed in the VS Code IDE.

Regarding the specific experimental workflow, given the substantial computational demands introduced by the network structure improvements and data augmentation operations, a stepwise development and deployment strategy was adopted. Early-stage code logic verification, module debugging, and small-batch inference testing were performed locally on a single NVIDIA GeForce RTX 1650 GPU. After the successful of local testing was confirmed and the loss function converged properly, the complete dataset and training scripts were transferred to a high-performance server equipped with dual NVIDIA GeForce RTX 3090 GPUs (each with 24 GB of memory) for full-scale training.

Furthermore, the algorithm development and experiments in this study were performed with a locally deployed Ultralytics 8.4.25 framework (Liu et al., 2026). Leveraging the mature industrial-grade operators and efficient training protocols of this framework, the improved modules were seamlessly embedded as plugins by modifying the source code of the core library. This strategy ensured that all ablation experiments were performed under a unified benchmark environment, thereby guaranteeing the fairness and scientific validity of the comparative experiments from the bottom up. The specific hardware and software parameters are detailed in **Table 1**.

**Table 1:** Experimental hardware and software parameters.

Environment type	Configuration item	Specific parameters/version
Hardware (Local)	GPU	NVIDIA GeForce RTX 3060 (12GB)
	CPU	Intel Core i7-12700K
	RAM	16GB
Hardware (Server)	GPU	NVIDIA GeForce RTX 3090 (24GB) × 2
	CPU	Intel Xeon Silver 4210
	RAM	128GB
Software	Operating System	Ubuntu 22.04 LTS
	Deep Learning Framework	PyTorch 2.5.1+cu121
	Core Algorithm Library	Ultralytics 8.4.25
	Language & Tools	Python 3.10.20, Conda, VS Code
	Parallel Computing Platform	CUDA 12.1, cuDNN 8.9.x

To ensure the reliability and reproducibility of the experiments, the model parameters were strictly limited during training. The input image resolution was uniformly set to 800 pixels ×800 pixels to retain the fine edges of the SSs. The total number of training iterations was set to 200, the batch size was set to 32, and a 50-round early stopping mechanism was introduced to prevent overfitting. In terms of data augmentation, the probability of the mosaic strategy was set to 0.8 and the probability of the mix-up strategy was set to 0.4. The random scaling factor was limited to 0.3 to prevent shape distortion. In terms of optimization strategies, the AdamW optimizer was selected, with an initial learning rate of 0.0001, and the cosine annealing strategy was used for scheduling. The weight decay coefficient was set to 0.01 to increase generalizability. Additionally, owing to the lack of instance segmentation pretrained models in the agricultural field, the general rtdetr-l detection weights were used as the base of backbone network, while the segmentation prediction head was randomly initialized and fine-tuned during training.

#### 2.2.6 Definition of evaluation metrics

To objectively evaluate the detection and segmentation performance of the improved model for densely distributed SSs in a complex sugarcane farmland environment, in this paper, the multidimensional selects the multi-dimensional mean average precision (mAP), mean absolute error (MAE), and number of frames per second (FPS) were selected as the core evaluation indicators (Lin et al., 2014).

##### 2.2.6.1 mAP

Considering that both the bounding boxes of the targets and the pixel-level masks must be output in the instance segmentation task, the bounding box detection accuracy (mAP^box^) and the mask segmentation accuracy (mAP^mask^) were selected to evaluate the model’s macroscopic positioning and microscopic segmentation capabilities, respectively. These indicators were calculated on the basis of the precision (P) and recall (R), and the specific calculation formulas are shown in (17) and (18), respectively in Equations 23, 24:

(23) $$\text{P}=\frac{\text{TP}}{\text{TP}+\text{FP}}$$

(24) $$\text{R}=\frac{\text{TP}}{\text{TP}+\text{FN}}$$

In the above expression, TP represents the number of individual SSs that were successfully and accurately segmented by the model, FP indicates the background noise targets that were incorrectly identified as targets, and FN describes the number of SSs that were not detected and thus not recognized by the system. By combining the precision and recall results, the average precision (AP) and mAP can be derived. Considering that this study focuses only on a single target (category number “N=1”), namely, SSs, the mAP (Equation 26) is numerically equivalent to the AP (Equation 25):

(25) $$\text{AP}=\int_{0}^{1}\text{P}(\text{R})\text{dR}$$

(26) $$\text{mAP}=\frac{1}{\text{N}}\sum_{\text{i}=1}^{\text{N}}{\text{AP}}_{\text{i}}$$

To fully verify the effectiveness of the improved algorithm, in this paper, the following three evaluation metrics were selected: ${\text{mAP}}_{50}^{\text{box}}$: This metric represents the bounding box detection accuracy when the IoU threshold is 0.5. This metric measures the model’s ability to capture the approximate position of individual sugarcane targets in scenes with complex backgrounds. ${\text{mAP}}_{50}^{\text{mask}}$: This metric represents the mask segmentation accuracy when the IoU threshold is 0.5, reflecting the model’s pixel-level decoupling ability for adhered contours. ${\text{mAP}}_{50-95}^{\text{mask}}$: This indicator requires calculating the average accuracy of the mask at 10 points with IoU thresholds ranging from 0.to 0.95 (with a step size of 0.05), and then taking the average value. It has more stringent requirements for the overlap between the predicted mask and the actual physical boundary.

##### 2.2.6.2 MAE

The stems and leaves of sugarcane plants in the field often overlap, which frequently leads to single SSs being counted as multiple instances because their growth is blocked by the leaves. Therefore, the MAE metric is introduced to measure the deviation between the total number of SSs counted by the model and the manually verified GT value. The calculation formula is as follows in Equation 27:

(27) $$\text{MAE}=\frac{1}{\text{N}}\sum_{\text{i}=1}^{\text{N}}\left|{\text{C}}_{\text{i}}-{\hat{\text{C}}}_{\text{i}}\right|$$

where N represents the total number of images in the test set, and C_i_ and ${\hat{\text{C}}}_{\text{i}}$ denote the GT and number of SSs predicted by the model in i-th image, respectively. The MAE intuitively reflects the absolute difference between the predicted value and the GT. A lower value indicates that the model can betterdeduplicate targets and avoid missed detections of SS under DO conditions.

##### 2.2.6.3 Inference Speed Metric (FPS)

To evaluate the real-time deployment potential of the model, this study adopts FPS was adopted to measure the efficiency of the proposed algorithm. This metric is calculated as follows:

(28) $$\text{FPS}=\frac{1}{{\text{T}}_{\text{avg}}}$$

where T_avg_ represents the average time required for the network to process a single image. Generally, a higher FPS value implies better real-time application potential for the algorithm on low-power edge devices.

## 3 Results

### 3.1 Performance evaluation metrics of the baseline model

**Figure 12:**
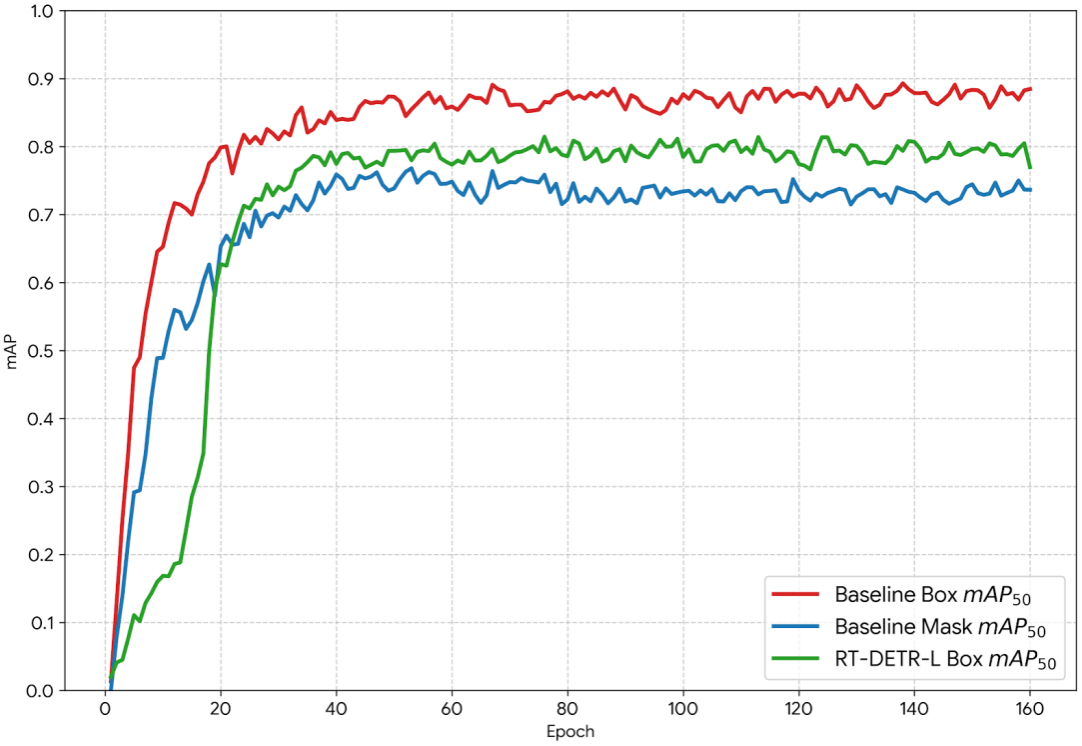
Comparison curve of the improvement in the mAP@0.5 metric between the pure detection and segmentation baseline models.

Considering that the preset maximum number of training iterations is two hundred rounds, an early stopping mechanism was preconfigured within the system to prevent the risk of overfitting; therefore, when the key accuracy indicators of the validation set failed to significantly improve after nearly one hundred iterations, the training process was automatically terminated by the system after 163 rounds. At this point, all the loss curves had reached a smooth and stable state, which is sufficient to indicate that the baseline model has fully converged and that the obtained set of weights can serve as the basis for the subsequent ablation experiments. After the model fully converges, the node weights with the best performance on the validation set (the 76th round) were selected to conduct the final evaluation on an independent test set containing 126 images. Additionally, to explore the feedback effect of the mask segmentation branch in the multitask learning architecture on the detection accuracy, a pure detection architecture (RT-DETR-L) was specially added as a reference. The increase in the precision of the two models during training stage is shown in **Figure 12**, and the specific evaluation indicators on the test set are compared in **Table 2**.

**Table 2:** Evaluation metrics of the original RT-DETR-seg baseline model on the test set.

Model	$mAP_{50}^{box}\left(\%\right)$	${\text{mAP}}_{50}^{\text{mask}}\left(\%\right)$	${\text{mAP}}_{50-95}^{\text{mask}}\left(\%\right)$	MAE	FPS (RTX3090)
RT-DETR-seg (Baseline)	88.08	75.79	28.23	1.09	87
RT-DETR-L	80.84	–	–	1.24	114

The data in **Table 2** indicate that the introduction of the instance segmentation branch produces a significant positive feedback for the target detection task. The bounding box detection accuracy of the RT-DETR-seg model reaches 88.08%, which is nearly 7% greater than that of the pure detection network RT-DETR-L. The convergence trends of the two models are shown in **Figure 12**, revealing that the multitask combined architecture not only increases the maximum accuracy of the model, but also significantly increases the convergence speed in the early training stage. The underlying logic for this phenomenon is that in the operation environment, where the sugarcane targets severely overlap and the background is extremely chaotic, the pixel-level mask supervision signal prompts the backbone network to better filter out environmental noise during feature extraction, focusing on the contour features of the sugarcane itself, thereby assisting the detection head in achieving more accurate box positioning. Compared with the excellent detection performance, the mask segmentation ability of the baseline model has obvious limitations. As shown by the green curve in **Figure 12**, the ${\text{mAP}}_{50}^{\text{mask}}$ of the model ultimately stabilized at 75.79%. The precision gap between the detection and segmentation results highlights the limitations of the conventional segmentation head when dealing with specific agricultural and forestry targets. Specifically, when facing the slender geometric shape of sugarcane targets and the frequent lateral leaf occlusion, existing networks are prone broken to features, and ensuring the integrity of the mask is difficult.

In conclusion, the original model cannot handle fine segmentation tasks in complex farmland environments. To address these deficiencies, in the following sections ASCs are introduced to enhance longitudinal features, the long-range dependency CA mechanism is constructed to establish spatial connections across occlusions, and a loss function that integrates geometric and light robustness is designed to strictly constrain the mask boundaries.

### 3.2 Ablation experiments on the improved modules

To verify the effectiveness of the proposed ASC, CA mechanism, and joint MGR loss function incorporating geometric constraints and photometric robustness in the SS instance segmentation and counting task, ablation experiments were conducted under a unified hardware platform and hyperparameter settings. The experiments followed the controlled variable method, and the original RT-DETR-seg was used as the baseline model. In all the ablation experiments the same set of pretrained weights was used during training, and the improved modules were introduced progressively, with the aim of quantitatively evaluating the specific contribution of each innovative module to model performance.

#### 3.2.1 Quantitative comparison of the performance metrics

The evaluation results of the ablation experiments are shown in **Table 3**. The experimental evaluation metrics included the bounding box detection precision ${\text{mAP}}_{50}^{\text{box}}$, mask segmentation precision ${\text{mAP}}_{50}^{\text{mask}}$ and ${\text{mAP}}_{50-95}^{\text{mask}}$ counting accuracy (MAE), as well as number of model parameter (Params) and inference speed (FPS).

**Table 3:** Comparison of the evaluation metrics in the ablation experiments with the improved modules.

Model	Params (M)	$mAP_{50}^{box}\left(\%\right)$	${\text{mAP}}_{50}^{\text{mask}}\left(\%\right)$	${\text{mAP}}_{50-95}^{\text{mask}}\left(\%\right)$	MAE	FPS (RTX3090)
Baseline	43.5	88.08	75.79	28.23	1.09	87
+ ASC	45.1	89.12	78.85	32.37	0.62	82
+ ASC + CA	45.2	89.54	79.51	33.8	0.35	81
Final	45.2	91.03	81.04	34.88	0.28	81

The results presented in **Table 3** indicate that as each improved module is sequentially introduced into the model, the overall performance of the model in complex sugarcane field environments tends to steadily improve.

##### 3.2.1.1 Analysis of the effectiveness of the ASC

The detection and segmentation accuracy of the model increased by 1.04% and 2.43%, respectively, when the ASC module was introduced into the baseline model. This finding indicates that the ASCs can better fit the physical characteristics of the SSs, which not only enhances the model’s ability to aggregate longitudinal features but also effectively reduces lateral background interference. Moreover, the model’s MAE decreased from 1.09 to 0.62, and the number of parameters only increased by only 1.6 M, which ensures that the improved model can still be feasibly deployed on edge devices.

##### 3.2.1.2 Analysis of the effectiveness of the CA mechanism

After the CA mechanism was embedded on top of the ASC module, the model achieved an ${\text{mAP}}_{50-95}^{\text{mask}}$ of 33.80%, representing an improvement of 1.43% compared with that in the previous stage for this key metric. This finding indicates that the CA mechanism successfully establishes LRS dependencies across the network’s deep layers by decoupling and encoding the spatial information of the feature maps in both the horizontal direction and the vertical direction. This mechanism effectively alleviates the problem of duplicate counting caused by instance ID fragmentation, and the MAE was further optimized and reached 0.35.

##### 3.2.1.3 Analysis of the effectiveness of the MGR loss

After the final model was constructed using the MGR combined loss function, all the evaluation indicators reached the optimal values. Specifically, the bounding box detection accuracy ( ${\text{mAP}}_{50}^{\text{box}}$) was 91.03%, the mask segmentation accuracy ( ${\text{mAP}}_{50}^{\text{mask}}$) reached 81.04%, and the MAE decreased to 0.28. These data indicate that the MGR loss function effectively guides the network to fit the true physical shape of the SSs. In field scenes with complex light and shadow changes, the geometric constraints inherent in this loss function can significantly suppress the distortion and expansion of the predicted mask edges, thereby successfully decoupling the mask adhesion between dense SS and ultimately achieving high-precision single-stem SC.

#### 3.2.2 Training process evaluation

**Figure 13:**
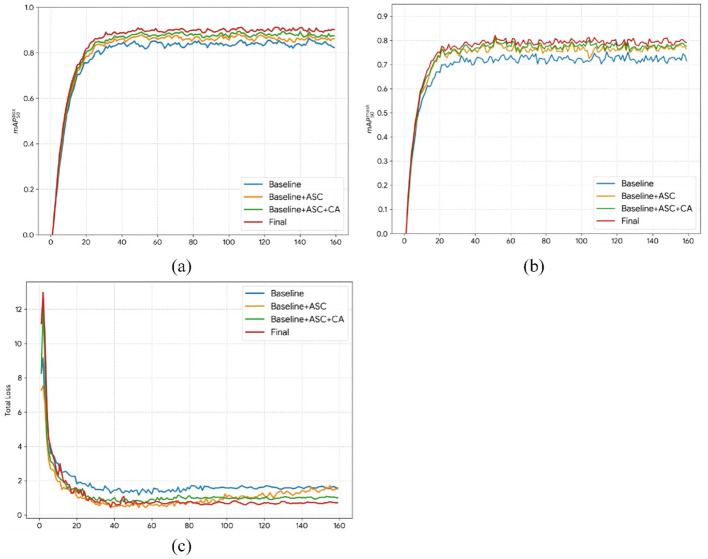
Comparison of the dynamic trends of the accuracy improvement and loss convergence for each ablation model. **(a)** Curves of the improvement in ${\mathrm{mAP}}_{50}^{\mathrm{box}}$; **(b)** Curves of the improvement in ${\mathrm{mAP}}_{50}^{\mathrm{mask}}$; and **(c)** Total loss convergence curve on the validation set.

To further analyze the impact of the model improvements on model convergence, the accuracy improvements and loss convergence of the four ablation models on the validation set were compared, as shown in **Figure 13**. The results in **Figures 13a, b** reveal that compared with that of the baseline model, the gradient of the final model increases sharply in the early training stage, indicating that the improved feature extraction network can more quickly capture the discriminative semantic features of the sugarcane targets. Furthermore, the loss curve in **Figure 13c** reveals that although the final model introduces additional geometric penalty terms (L_ar_ and L_smooth_) which result in a higher initial loss value, its convergence lower bound is significantly lower than that of the baseline model, and the overall decline in the loss is smoother. This high-penalty, high-accuracy convergence pattern clearly demonstrates that the joint loss function constructed in this paper can effectively guide the physical prior, thereby prompting the network weights to be updated rapidly towards the optimal values with clear physical meaning.

#### 3.2.3 Feature response analysis

Next, the network’s ability to extract coherent semantic features across occlusions was verified at the feature level, and the smoothness-constraining effect of the joint loss function on the mask geometry was intuitively demonstrated through the final prediction results.

##### 3.2.3.1 Cross-occlusion feature response analysis

**Figure 14:**
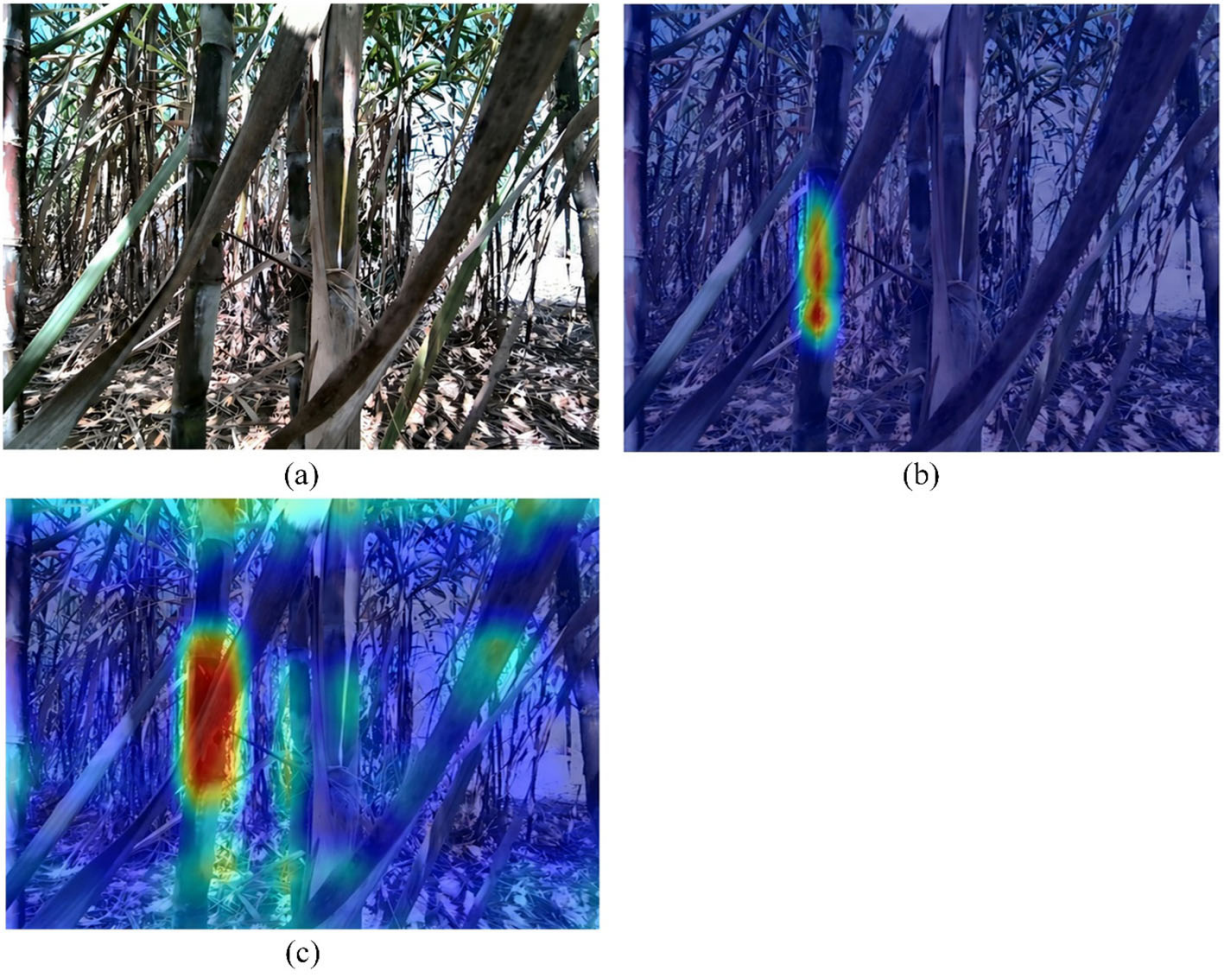
Comparison of the feature response heatmaps between the baseline model and the improved model under severe occlusion scenarios. **(a)** Original image; **(b)** baseline model heatmap; and **(c)** improved model heatmap.

In this study, Grad-CAM was employed to extract feature response heatmaps of the baseline model and the final model under typical SS occlusion scenarios (**Figure 14**). A comparison of the data in **Figure 14b** reveals that when the baseline model faces the issue of physical truncation of the SSs by dead leaves in the horizontal direction, the high-response regions of the SSs exhibit significant discontinuity at the occlusion boundaries. This reflects that owing to the relatively limited spatial sampling field of view, the baseline network struggles to maintain the integrity of the target semantic features in the case of discontinuous visual signals, and a single SS is easily identified as fragmented feature segments, thereby resulting in redundant counts.

A comparison of the data in **Figure 14c** clearly reveals that the improved model successfully addresses the occluding dead leaves of the SSs, and a coherent longitudinal bright band is formed. Furthermore, while the baseline model’s response is confined to the central region, the improved model simultaneously activates other SSs in the background. This finding demonstrates that the ASC module enhances longitudinal feature extraction, whereas the CA mechanism expands the global receptive field, enabling the network to use the consistency of the spatial coordinates to reassociate discontinuous SS features and identify dense SSs.

##### 3.2.3.2 Qualitative mask comparison

**Figure 15:**
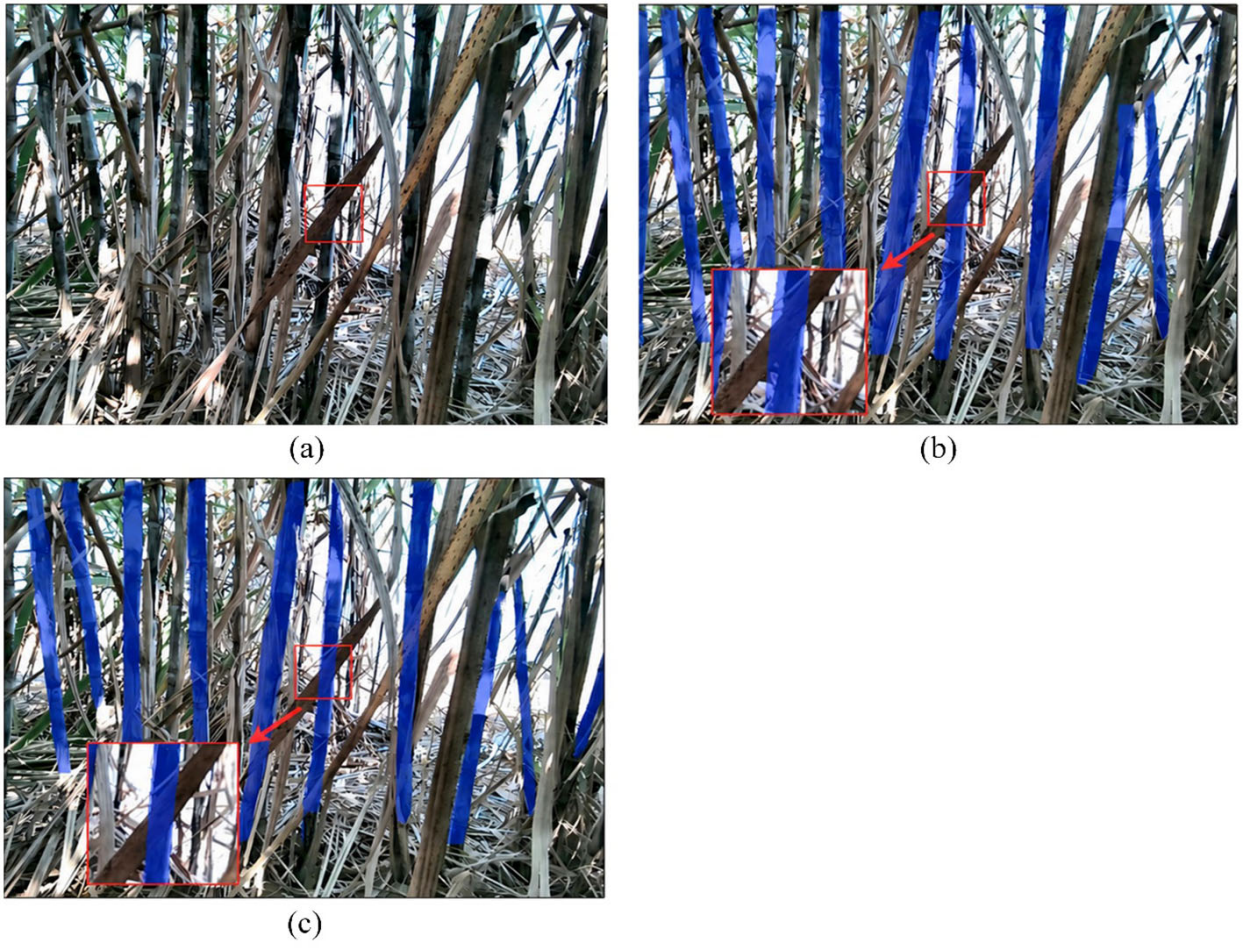
Qualitative comparison of the smoothness of the mask based on the MGR loss under complex lighting and shadow conditions. **(a)** Original image; **(b)** localized zoomedin view of the prediction results of the baseline model; and **(c)** localized zoomed-in view of the prediction results of the improved model.

To further verify the macroscopic geometric constraint effect of the MGR loss function, the mask prediction results of the model under mottled shadow and dead leaf occlusion conditions are extracted for qualitative comparison (**Figure 15**). As shown **Figure 15b**, before the MGR constraint is introduced, the mask edges, which are influenced by the local pixel-level loss, exhibit obvious nonphysical fluctuations in the prediction stage and are very prone to severe adhesion with background shadows. After the MGR loss is introduced, owing to the strict penalties on the second-order spatial moments and gradient magnitude, the predicted boundaries are significantly straightened and smoothed and precisely fit the inherently straight geometric contour of the main SS.

### 3.3 Comparison experiments

To objectively evaluate the overall performance of the improved RT-DETR-seg model, representative single-stage instance segmentation algorithms were selected for horizontal comparison experiments. All the models included in the comparison were trained and tested on the same sugarcane multiscene dataset described in Section 2.1.1 (“Image Acquisition Parameters and Construction of Complex Scenarios”), with identical hardware environments and parameter settings used to ensure the rigor of the evaluation results.

#### 3.3.1 Experimental setup and baseline models

In this study, mainstream YOLO series algorithms were introduced to perform comparative experiments to construct a multidimensional performance reference. Specifically, YOLOv8l-seg was selected to benchmark the performance of the CNN in general segmentation tasks, while the large (l) and lightweight (n) versions of YOLOv11 were used to explore the maximum accuracy of the single-stage models and their potential for edge-side applications, respectively. These types of models are inherently limited by their local feature extraction fields and the NMS mechanism and thus often face detection bottlenecks under field conditions such as high sugarcane overlap and frequent leaf occlusions. Through such comparisons, the adaptability and robustness of our end-to-end transformer architecture in extreme environments can be intuitively verified.

#### 3.3.2 Comparison of the evaluation metrics

The evaluation results of each mainstream model on the test set are shown in **Table 4**. To illustrate the dynamic characteristics of different architectures during training, the detection and segmentation performance of each model on the validation set are plotted in **Figure 15**.

**Table 4:** Performance comparison of the instance segmentation algorithms on the sugarcane test set.

Model	Params (M)	$mAP_{50}^{box}\left(\%\right)$	${\text{mAP}}_{50}^{\text{mask}}\left(\%\right)$	${\text{mAP}}_{50-95}^{\text{mask}}\left(\%\right)$	MAE	FPS (RTX3090)
YOLOv8l-seg	45.9	90.67	82.20	33.79	1.16	102
YOLOv11n-seg	2.9	89.68	80.03	32.45	1.75	315
YOLOv11l-seg	27.6	90.72	82.25	35.11	1.13	134
Ours	45.2	91.03	81.04	34.88	0.28	81

##### 3.3.2.1 Analysis of the bounding box detection precision

**Figure 16:**
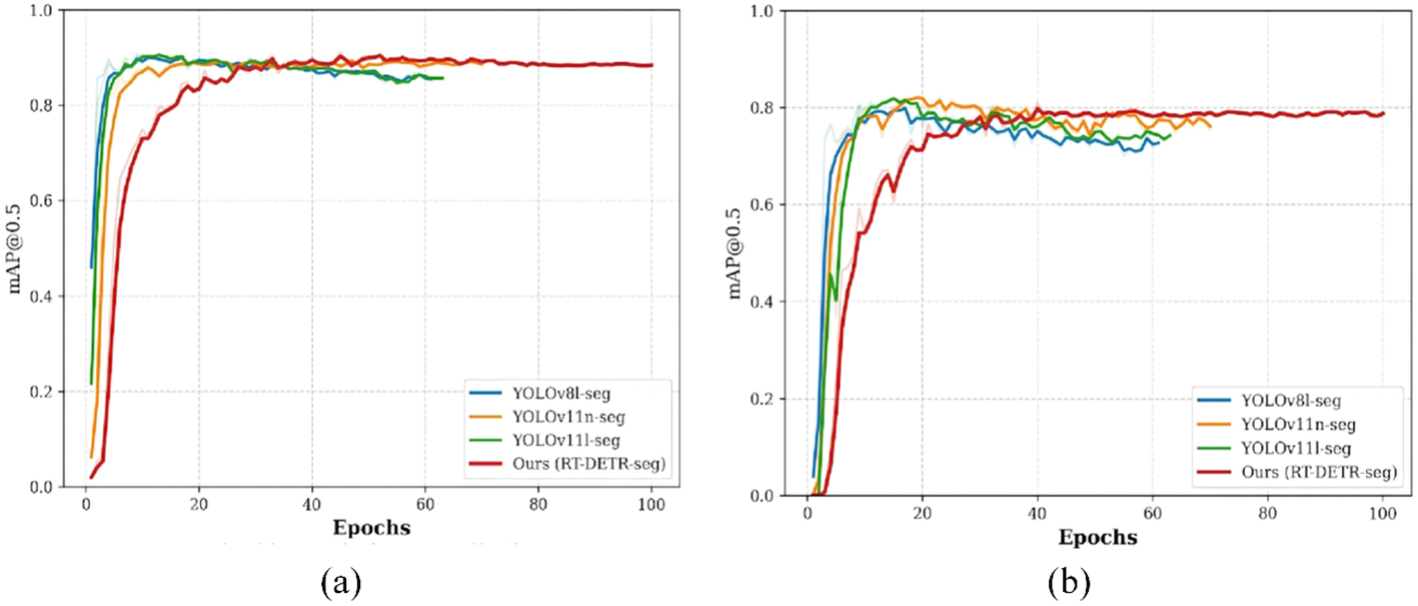
Comparison of the detection accuracy and segmentation accuracy of each model during training. **(a)** Object detection accuracy; and **(b)** mask accuracy and ${\mathrm{mAP}}_{50}^{\mathrm{mask}}$.

The data in **Table 4** and **Figure 16a** reveal that our model achieves the best performance among the various algorithms in terms of the bounding box detection precision ${\text{mAP}}_{50}^{\text{box}}$ with a value of 91.03%. In terms of the convergence process during the training phase, because of their inherent advantage of local inductive bias, the convolutional networks, represented by YOLOv11l-seg, converge quickly within the first 20 epochs; however, the curves exhibit notable oscillations during the later stages of training. In contrast, the increase in the precision of our model is relatively smooth. Although the transformer architecture lacks prior bias information, causing its convergence speed to be slower than that of the convolutional networks in the early stage, after 40 epochs, owing to the effective extraction of longitudinal geometric features by the ASC module, our model’s precision steadily exceeds that of the YOLO series models, and the precision of our model is both higher and more stable in the final stage of training.

##### 3.3.2.2 Fundamental differences in the counting accuracies (MAE) of the models

For visual counting tasks in the field, the conventional detection accuracy and segmentation accuracy often fail to fully reflect the true performance of the model. The experimental results show that although the mask accuracy of YOLOv11l-seg is approximately 1.2% greater than that of the proposed model, its MAE for counting reaches 1.13. The main reason for this error lies in the inherent disadvantage of convolutional networks when dealing with physical truncations. Specifically, when the SSs are occluded by leaves and visual breaks occur, owing to the local receptive field and NMS postprocessing mechanism, the YOLO algorithm is prone to mistakenly identifying the disconnected fragments of the same plant as multiple independent targets. To solve this problem, the proposed model uses the CA mechanism to explicitly encode the row and column positions in the global space, thereby establishing long-range dependencies across the occlusion area. This mechanism enables the network to logically reassociate the broken SS, thereby effectively avoiding duplicate counting, and significantly reducing the MAE to 0.28. Moreover, as shown in **Figure 16b**, the accuracy of the proposed model in the later training stage is very stable, which confirms its high stability in the counting task.

##### 3.3.2.3 Operational efficiency and engineering applicability

Under the same hardware conditions (RTX 3090), the inference frame rate of the lightweight YOLOv11n-seg network is as high as 315 FPS. However, the relatively high counting error makes it difficult to meet the actual yield measurement requirements in the field. In contrast, after the CA mechanism and geometric constraints are added, the proposed model has only 45.2 M parameters and an inference speed of 81 FPS.

Owing to the large computational overhead of the self-attention mechanism in the transformer architecture, the speed of the proposed model is not as fast as that of YOLOv8l-seg (102 FPS) and YOLOv11l-seg (134 FPS), which have the same number of parameters. However, despite this moderate speed, the accuracy is significantly increased, and the MAE is reduced from 1.09 to 0.28. Considering the complex field environment of sugarcane and the high possibility of overlap, prioritizing the reliability of the counting results is more practical than simply pursuing an extremely high frame rates. Moreover, the benchmark speed of 81 FPS does not result in serious performance bottlenecks. Combined with acceleration methods such as TensorRT quantization, the proposed method can potentially smoothly on edge devices such as Jetson.

## 4 Discussion

**Figure 17:**
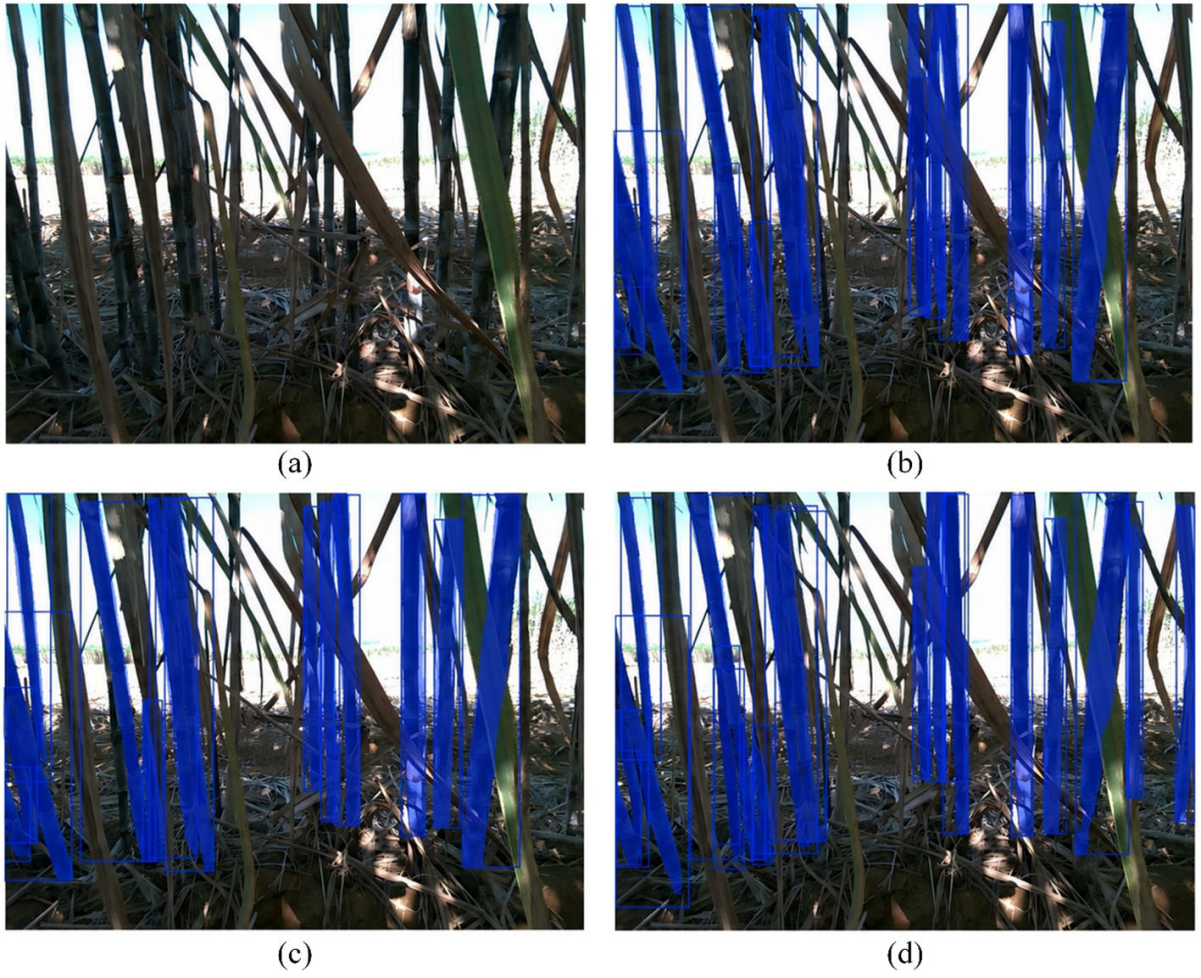
Comparison of the instance segmentation effects of different algorithms in dense occlusion scenarios. **(a)** Original input image; **(b)** YOLOv8l-seg result (16 stalks); **(c)** YOLOv11l-seg result (14 stalks); and **(d)** improved model result (17 stalks).

To further investigate the sources of counting errors, a DO sample containing 17 SSs was selected for visual verification. When dealing with areas where the SSs are closely arranged or covered by dead leaves in the lateral direction, obvious instance connection problems were observed with the CNN model. Owing to the limited field of view of the local extraction window, the algorithm was prone to mistakenly identifying adjacent independent stems as the same entity. Moreover, owing to the duplicate suppression logic in the NMS, the model stage was prone to mistakenly identifying overlapping actual targets as redundant frames and thus filtering them out, directly leading to missed detections in YOLOv8l-seg and YOLOv11l-seg, resulting in the final identified numbers (16 stalks and 14 stalks respectively) being lower than the actual numbers (**Figure 17**).

The proposed model effectively addresses the boundary confusion between dense SS targets by introducing global context modelling and longitudinal feature enhancement strategies. In areas with severe physical occlusion among SSs, the model still relies on global coordinate anchoring to maintain the semantic independence of different sugarcane targets, with clear mask division. Finally, all 17 SS targets were accurately counted. This visual phenomenon is consistent with the quantitative data described above, confirming the improvements of the proposed modules in terms of suppressing missed detections and increasing the counting accuracy. The specific prediction mask effects of each algorithm in the DO scenarios are shown in **Figure 17**.

In this paper, a systematic performance evaluation of the improved RT-DETR-seg model was conducted on the basis of a multiscenario SS dataset. The baseline test results clearly indicate the limitations of the original architecture in handling the elongated shape, DOs, and complex lighting conditions of sugarcane, which lead to feature breakage and edge distortion. The ablation experiments confirm the actual improvements of each proposed module: under the combined effects of the ASC module, CA mechanism, and MGR loss function, the model’s bounding box detection accuracy ( $mAP_{50}^{box}$) increases to 91.03%, the mask segmentation accuracy ( $mAP_{50}^{mask}$) reaches 81.04%, and the MAE significantly decreases from the 1.09 score of the baseline model to 0.28. Moreover, the comparison results with mainstream single-stage algorithms such as YOLOv8l-seg and YOLOv11l-seg show that the proposed model effectively overcomes the problem of repeated counting caused by physical truncation while maintaining an inference frame rate of 81 FPS.

Although relatively satisfactory results were obtained with the proposed sugarcane visual counting methods, limitations regarding the research time and hardware conditions should be considered. This system still has certain limitations in actual large-scale agricultural applications. In the future, more in-depth research should be performed to address the following aspects:

The proposed algorithm relies mainly on RGB optical images for feature extraction. In future research, the depth information provided by the D435i camera can be fully utilized or a low-cost single-line laser radar system can be introduced to construct an RGB-D multimodal fusion perception network. By leveraging physical depth information to decouple the foreground sugarcane and background weeds in three dimensions, the detection robustness of the algorithm under extreme occlusion and night lighting conditions can be greatly increased.

Additionally, the established dataset focuses mainly on specific varieties of sugarcane at the middle and late growth stages. However, the stem node colors and shape ratios of different varieties of sugarcane may differ, and the canopy structures may differ in different growth periods, such as the seedling stage and the tillering stage. In subsequent work, the scale of the field dataset should be expanded, or domain adaptation and other transfer learning techniques should be employed to increase the generalizability of the model across regions and growth periods.

## 5 Conclusions

To address the problems of low efficiency and high error rates in manual SC caused by dense stem distribution and severe leaf occlusion during the mid-to-late growth stages, an end-to-end instance segmentation method based on deep learning for in-field SS counting in unstructured agricultural environments was systematically investigated.

A multiscene SS with instance segmentation dataset was collected in the field and processed. A total of 1–264 original images containing various hard examples such as strong lighting and backlighting conditions, were obtained. Given the elongated morphology and overlapping characteristics of SSs, the polygon tool was used for precise, pixel-level, homogeneous instance annotation. Furthermore, data augmentation strategies, including the CLAHE, mosaic, and mix-up schemes, were employed to effectively increase data diversity, providing high-quality data support for model training.

A high-precision in-field SS with instance segmentation algorithm based on an improved RT-DETR-seg model was proposed. To address the issue of instance feature fragmentation caused by the elongated morphology of the SSs and complex occlusions, the ASC module was introduced into the backbone network to enhance vertical feature extraction and suppress background noise.

Additionally, the CA mechanism was embedded in the hybrid encoder to reconstruct LRS dependencies across occlusion areas, thereby effectively alleviating repeated counting errors. Moreover, the MGR loss, which integrates spatial moments and gradient field constraints was designed to increase the smoothness of the edges under strong light and shadow interference conditions.

Furthermore, the performance of the improved RT-DETR-seg model was systematically evaluated on the basis of the multiscenario SS dataset. The baseline test results clarified the limitations of the original architecture in terms of handling the slender morphology, Dos, and complex lighting conditions of SSs, leading to stem feature breakage and edge distortion. The ablation experiments confirmed the improvements of each proposed module: under the collaborative effect of the ASC module, CA mechanism, and the joint MGR loss function, the model’s bounding box detection accuracy ( $mAP_{50}^{box}$) increased to 91.03%, the mask segmentation accuracy ( $mAP_{50}^{mask}$) reached 81.04%, and the MAE significantly decreased from the 1.09 score of the baseline model to 0.28. Overall, the improved algorithm balances robust perception and real-time inference under complex field conditions, providing a solid algorithmic foundation for the development and deployment of edge-end SS counting systems.

## Data Availability

The data analyzed in this study is subject to the following licenses/restrictions: The datasets generated and/or analyzed during the current study are not publicly available but are available from the corresponding author on reasonable request. Requests to access these datasets should be directed to yuanjia.ma@gdupt.edu.cn.
